# Advancing time-since-interval estimation for clandestine graves: a forensic ecogenomics perspective into burial and translocation timelines using massively parallel sequencing

**DOI:** 10.3389/fmicb.2025.1684366

**Published:** 2025-11-14

**Authors:** Cherene de Bruyn, Kirstie Scott, Heather Panter, Frederic Bezombes, Komang Ralebitso-Senior

**Affiliations:** 1 Forensic Research Institute, Liverpool John Moores University, Liverpool, United Kingdom; 2 School of Pharmacy and Biomolecular Sciences, Faculty of Health, Innovation, Technology and Science, Liverpool John Moores University, Liverpool, United Kingdom; 3 School of Biological and Environmental Sciences, Faculty of Health, Innovation, Technology and Science, Liverpool John Moores University, Liverpool, United Kingdom; 4 School of Law and Justice Studies, Faculty of Society and Culture, Liverpool John Moores University, Liverpool, United Kingdom; 5 General Engineering Research Institute, Faculty of Health, Innovation, Technology and Science, Liverpool John Moores University, Liverpool, United Kingdom

**Keywords:** clandestine burials, soil microbiome, time-since-interval, post-mortem interval, post-burial interval, post-translocation interval, massively parallel sequencing, forensic ecogenomics

## Abstract

Forensic taphonomy and entomology has focused on estimating the post-mortem interval (PMI), particularly for surface depositions, using human cadavers and other mammalian models by considering morphological changes of the body and insect activity during decomposition. The PMI is crucial in forensic investigations as it provides key information regarding the victim’s identity, the circumstances of their death and can confirm or refute a suspect’s alibi. Gravesoil microbial communities are a potential tool that can complement traditional approaches to detect and confirm the presence of human remains in clandestine burials, aiding forensic investigations. The estimation of the time-since-burial (post-burial interval; PBI), and the time-since-translocation (post-translocation interval; PTI), a new concept, have potential to aid clandestine grave location but have received relatively little attention in forensic ecology research. Advances in massively parallel sequencing (MPS) provide a high-throughput means to estimate PBI and PTI by characterising soil microbial communities in graves with remains, from early to skeletal stages of decomposition, or where remains have been intentionally removed from crime scenes and relocated. This review presents a perspective on the use of the soil microbiome as an indicator for post-mortem time-since-interval estimations, with specific focus on the PBI and PTI. In addition, it provides a framework, supported within forensic ecogenomics, on how the PBI and PTI can be used as a forensic tool complemented by MPS. The review highlights the need for further research to validate microbial community analysis across diverse biogeographical regions to enhance its precision and reliability as a forensic investigative tool. Such validation could potentially enhance the accuracy of post-burial and post-translocation interval estimations, ultimately improving methods for clandestine grave identification.

## 1 Introduction

The timeline of events prior to and after the death of an individual can provide crucial information to forensic investigators. The information can include, but is not limited to: victim identification, time of death estimation, crime scene reconstruction, and confirming or refuting a suspect’s alibi (Cockle and Bell, 2015). For this reason, extensive research using a range of approaches has been conducted focusing on predicting and reliably estimating the time-since-death or the post-mortem interval (PMI). The PMI is typically defined as the period from the death of an individual until the body is discovered (Wilson-Taylor and Dautartas, 2017). The estimation of the PMI has advanced in parallel with an understanding of the decomposition process (Payne, 1965; Payne et al., 1968). Temperature has traditionally been viewed as the primary catalyst for body decomposition (Mann et al., 1990; Vass et al., 1992; Bass, 1996; Megyesi et al., 2005). However, subsequent investigations involving mammalian cadavers revealed that while temperature is crucial for decay, it is not necessarily the primary factor driving the decomposition process. Instead, a wide range of biotic and abiotic factors have emerged as significant contributors to the mammalian decomposition process, both individually and in various combinations. Several living components (biotic) [such as insects (Rodriguez, 1982; Ames and Turner, 2003; Matuszewski and Mądra-Bielewicz, 2022), arthropods (Goff et al., 1988; Singh et al., 2018; Bonacci et al., 2021), vertebrate scavengers (DeVault et al., 2004; Young, 2017; Spies, 2022; Adams et al., 2024), fungi (Sagara, 1975; Carter and Tibbett, 2003; Gemmellaro et al., 2023), and microbial communities (Carter et al., 2007; Metcalf et al., 2013; Pechal et al., 2014; Hauther et al., 2015)] and non-living variables (abiotic factors) [such as soil pH (Haslam and Tibbett, 2009), temperature (Archer, 2004; Laplace et al., 2021), soil conditions (Haslam and Tibbett, 2009; Carter et al., 2010; Quaggiotto et al., 2019), burial conditions (Ahmad et al., 2011; Bhadra et al., 2014; Matuszewski et al., 2014; Martín-Vega et al., 2017; Pawlett et al., 2018; Cogswell and Cross, 2021; Bisker et al., 2024), weather/climate (Voss et al., 2009; Englmeier et al., 2023; Maisonhaute and Forbes, 2023) and individual characteristics of the victim (Mason et al., 2022)] can affect the body after death, impeding the forensic investigations.

**Figure 1:**
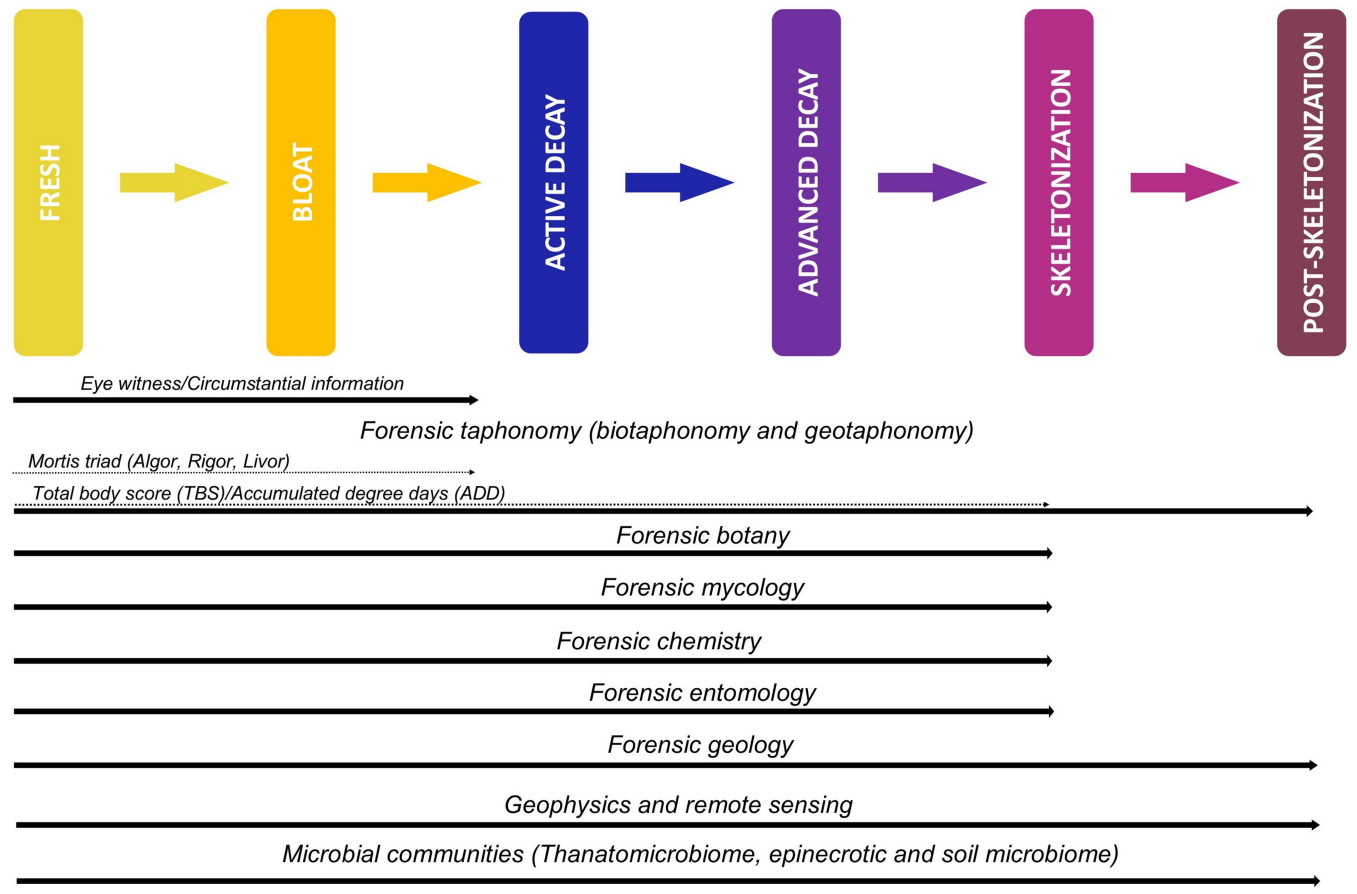
Decomposition timeline illustrating the post-mortem changes observed and the forensic subdisciplines used to aid in detecting remains and estimating the post-mortem and post-burial intervals. Adapted from Ralebitso-Senior and Olakanye (2018) and Metcalf (2019).

Several approaches (Figure 1) have been developed to assess the influence of biotaphonomic agents (such as environmental and climatic conditions, biotic factors and individual characteristics of the deceased like their body mass and height) and to monitor geotaphonomic changes (associated with the cadaveric processes and ground disturbances, including soil colour changes, changes in soil chemistry, changes in vegetation) resulting from the burial activity and decomposition process (Hochrein, 2002; Nawrocki, 2016; Prada-Tiedemann et al., 2024). These approaches have been used complementary to aid PMI estimation for remains across the decomposition timeline. Beyond the scope of estimating the PMI, forensic entomology (focusing on insect behaviour and succession during the decomposition process) (Rodriguez, 1982; O’Flynn, 1983; Micozzi, 1986; Galloway et al., 1989; Mann et al., 1990; Campobasso et al., 2001; Rai et al., 2022) vegetation growth patterns and forensic botany (use of vegetation in forensic investigations) (Watson and Forbes, 2008; Caccianiga et al., 2012), forensic mycology (use of fungi in forensic investigations) (Sagara, 1995; Carter and Tibbett, 2003; Bellini et al., 2016) and geophysical approaches (Pringle et al., 2020) have been used to locate human remains, clandestine burials and mass graves and additionally to estimate the time that has passed since a body was buried or deposited until it is discovered [the post-burial interval (PBI)] (Finley et al., 2015; Pringle et al., 2015; Singh et al., 2016).

While traditional approaches used to estimate the PMI and PBI are useful, they are nonetheless limited and largely applicable for estimating reliable time-since-intervals for remains in early decomposition stages (Henßge and Madea, 2004; Pittner et al., 2020; Laplace et al., 2021; Heinrich et al., 2024). For extended post-mortem intervals (remains in advanced stages of decay), these methods provide estimates with different margins of error because decomposition proceeds at a highly variable rate (Giles et al., 2022; Madea, 2023). In the case of forensic entomology, the PBI for advanced stages of decomposition is based on the succession of insects rather than oviposition and larval development (Campobasso et al., 2001). Succession patterns for remains in advanced decomposition or in prolonged desiccation are less precise because insect and arthropod diversity and abundance decrease over time as the remains become skeletonised and dry, which also leads to a reduction in nutrients and resource availability (Sharanowski et al., 2008; Rai et al., 2022). The ability of insects to colonise remains as well as vegetation to benefit from the release of nutrients from the body is further dependent on the treatment of the body and the burial depth (Rodriguez and Bass, 1985; VanLaerhoven and Anderson, 1999; Congram, 2008; Caccianiga et al., 2012; Pastula and Merritt, 2013; Bonacci et al., 2021). Additionally, due to the variability in burial conditions, environmental conditions and regional variation in species, further empirically validated studies to test the reliability of forensic entomology, forensic botany, and forensic mycology approaches in PBI estimation post-skeletonisation or in prolonged decomposition are needed (Coyle et al., 2005; Menezes et al., 2008; Sidrim et al., 2010; Bellini et al., 2016; Ventura et al., 2016; Watson et al., 2021). Even when biotic and abiotic factors are considered, the identification of victims of homicide and mass conflicts, and the estimation of the PMI and PBI by extension, become increasingly more challenging without their remains. Perpetrators can attempt to hide the victim’s remains by recovering them from their primary deposition site and intentionally reburying and concealing them at secondary locales (Karčić, 2017; Shapiro, 2020; Anstett, 2023).

**Table 2:** Summary of the methodological confounders and control measures in several studies applying microbial data for PMI estimation.

Study	Sample handling and extraction	Sequencing design and bioinformatics workflow	Diversity analysis, statistical treatment, model handling
Can et al. (2014)	Handling: Samples were transported in a cooler and placed in a freezer at −80 °C until analysis Extraction: Two extraction methods for thanatomicrobiome analysis: Modified method (Urakawa et al., 2010) using 50 μL of blood or approximately 10 mg of thawed organ tissue. Consists of bead-beating with phenol/chloroform/isoamyl alcohol precipitation A sterile cotton applicator tip was dipped into the organ and swabbed on the surface and the tip was deposited into a centrifuge tube containing 1 mL of PBS buffer	16S rRNA gene Platform: Roche 454 pyrosequencing Primers: - MG-RAST open-source web application Quality filtering Annotated against M5RNA database (contains data from SILVA, Greengenes and RDP) Normalization in MG-RAST	Diversity and statistical analysis: Shannon diversity Principle component analysis (PCA) on matrix on distances Clustering dendrograms of microbial communities by organ tissue and blood sample
Lauber et al. (2014)	Handling: Samples stored at −20 °C until further analysis Extraction: DNA extraction based on Earth Microbiome Project standard protocols and Metcalf et al. (2019) PowerSoil DNA isolation kit (MoBio Laboratories)	16S rRNA gene V4 region Platform: HiSeq 2000 (Illumina) Primers: 515F-806R QIIME Pipeline: Quality filtering Operational taxonomic unit (OTU) picking Reference database (Greengenes, 2012) Phylogenetic tree generation (PyNAST) Taxonomy assigned (RDP classifier) Normalisation: 3,000 sequences per sample	Diversity and statistical analysis: Alpha diversity using Faith’s phylogenetic diversity Beta diversity: PCoA with UniFrac unweighted distances PERMANOVA to test between treatments at each sample site
Pechal et al. (2014)	Handling: Samples were stored at 4 °C. Processing took place within 12 h of sampling Extraction: Modified chloroformphenol extraction	16S rRNA V1–3 regions Platform: Roche 454 FLX Titanium pyrosequencing Primers: Gray28F/Gray519R Pipeline: Non-bacterial ribosome sequence and chimera removal (B2C2) Taxonomic classification: (Ribosomal Database Project (RDP)) Taxonomic Classifier: Naïve Bayesian rRNA classifier version 2.2 in RDP	Diversity and statistical analysis: PERMANOVA for differences in taxon richness Bray–Curtis distance with nonmetric multidimensional scaling (NMDS) to analyse operational taxonomic units Multi-response permutation procedure (MRPP) to test differences between decomposition day and region of sampling of bacterial community composition Machine learning algorithm: Random forest Validation
Damann et al. (2015)	Handling: Ribs were individually bagged and transported on dry ice and stored at −20 °C until analysis. Soil samples were collected 5 cm below the remains, sieved through a 2 mm mesh, collected in a plastic bag, transported on dry ice, and stored at −20 °C until analysis Extraction: Bone: Modified demineralisation protocol with MinElute kit (Qiagen) (Loreille et al., 2007) Soil: Fast DNA Spin Kit for Soil (MP Biomedicals, UK)	16S rRNA gene V3 region Platform: GS FLX Titanium 454 pyrosequencing Primers: F338/R533 QIIME Pipeline: Chimera removal (UCHIME via USEARCH 6.1) OTU clustering at ≥97% similarity (UCLUST) Representative sequence alignment (pyNAST) Phylogenetic tree building with FastTree Classified (RDP) Normalization: 4000 reads per sample	Diversity and statistical analysis: UniFrac distances and PCoA for differences in microbial community membership and structure
Hauther et al., 2015	Handling: Swabs were collected in a sterile tube, stored on ice and stored at −20 °C until analysis Extraction: PowerSoil DNA Isolation Kit (MoBio Laboratories, Inc.)	16S rRNA gene *Bacteroides* with Taqmanâ qPCR assay, *Lactobacillus and Bifidobacterium* with SYBR Green PCR assay Quantification: qPCR (Opticon Monitor III/CFX96 (BioRad, Hercules, CA, USA)) Primers: Universal based on (Buchan et al., 2009) Normalisation: To total 16 s rRNA bacterial load	Diversity and statistical analysis: Linear and nonlinear models for best fit t-tests to determine if the variability between individual’s characteristics (body mass, sex, or cause of death) was significant
Javan et al. (2016a)	Handling: All samples were placed in a freezer at −80 °C until further analysis Extraction: By the phenol/chloroform method	16S rRNA gene V4 region Platform: MiSeq (Illumina) Primers: 515F/806R Pipeline: Denoising and chimera detection Clustering the reads into OTUs Taxonomic classification	Diversity and statistical analysis: Alpha diversity: Chao1 richness estimator and Shannon-Wiener diversity index Analysis of variance (ANOVA) to screen for microbial diversity for organ, manner of death, ethnicity, sex, age, PMI, and ambient temperature Multivariate differences among organ, manner of death, ethnicity, sex, age, PMI, and ambient temperature with Permutational Multivariate Analysis of Variance Using Distance Matrices function (ADONIS) PCoA to visualise relationships and differences between organ, manner of death, ethnicity, sex, age, PMI, and ambient temperature Machine learning algorithm: Random forest Validation
Johnson et al. (2016)	Handling: All samples were placed in a freezer at −80 °C until further analysis Extraction: PowerLyzer PowerSoil DNA Isolation Kit (MoBio Laboratory)	16S rRNA gene V3 and V4 regions Platform: MiSeq (Illumina) Primers: – Pipeline: BaseSpace program (Illumina). Normalisation: Column-based normalisation	Machine learning algorithm: Regressors: Support Vector Regression, K-neighbors Regression, Ridge Regression, Lasso Regression, Elastic Net Regression, Random Forest Regression, and Bayesian Ridge Regression Validation: Cross-validation on the training set
DeBruyn and Hauther (2017)	Samples were kept at −20 °C until further analysis PowerLyzer PowerSoil DNA Isolation Kit (MoBio Laboratory)	16S rRNA gene V3 and V4 regions Platform: MiSeq (Illumina) Primers: – Mothur (v.1.37.0) Pipeline: Chimaera removal (UCHIME) Sequences aligned (SILVA database) Clustering the reads into OTUs Taxonomic classification Normalisation: 25,082 sequences per sample	Diversity and statistical analysis: Alpha-diversity: Good’s coverage estimates, richness (number of OTUs), Simpson’s Diversity index Beta diversity Bray-Curtis distances between samples PERMANOVA to test significant differences in multivariate structure Non-parametric Spearman’s rank between the top 30 most abundant OTUs
Javan et al. (2017)	Handling: Samples were kept at −80 °C until further analysis Extraction: Lysing matrix E tubes (MP Biomedicals) with phenol/chloroform/isoamyl alcohol	16S rRNA gene V3 and V3-4 regions Platform: MiSeq (Illumina) Primers: 515F/806R (V3) and 357wF/785R (V3-4) PCR Control: Negative control reaction mix with not template DNA Sequencing: Denoising reads Chimaera removal (UCHIME) Clustering the reads into OTUs (UPARSE) Taxonomic alignment (USEARCH) Normalisation: 25,000 sequences per sample	Diversity and statistical analysis: Alpha diversity: Chao1 richness estimator and Shannon-Wiener diversityindex Analysis of variance (ANOVA) to screen for microbial diversity for region, organ, gender, manner of death, PMI, season, location, weight, and height Multivariate differences among region, organ, gender, manner of death, PMI, season, location, weight, and height with Permutational Multivariate Analysis of Variance Using Distance Matrices function (ADONIS) PCoA to visualise relationships and differences between region, organ, gender, manner of death, PMI, season, location, weight, and height
Pechal et al. (2018)	Handling: Samples were kept at −20 °C until further analysis Extraction: PureLink Genomic DNA Mini Kit (Invitrogen, USA)	16S rRNA gene V3 and V3-4 regions Platform: MiSeq (Illumina) Primers: 515F/806R QIIME Pipeline: Sequences clustered (UCLUST) Chimaera identification and removal (ChimeraSlayer) Taxonomy assignment: RDP classifier Identification: BLAST against Greengenes (2013) 97% reference Taxonomy alignment (PyNAST) Normalisation: 1,000 sequences per sample	Diversity and statistical analysis: Alpha-diversity: Chao1, Shannon-Wiener diversity, Heip’s evenness, and Faith’s phylogenetic diversity Beta diversity: PCoA with weighted UniFrac distance to examine umbilicus samples and analyse their differences in microbial communities PCoA to measure the significance of sex, ethnicity, event location, weight, season, manner of death, and PMI PERMANOVA to test differences in communities Nonparametric one-way analysis of variance (Kruskal-Wallis, ANOVA) with multiple comparisons after Mann-U t-tests to evaluate how diversity, richness and evenness changes after death Machine learning algorithm: Stochastic gradient boosting Validation: 10-fold cross-validation
Lutz et al. (2020)	Handling: Samples were transported on dry ice to Montgomery, USA and stored at −80 °C Extraction: Phenol phenol-chloroform method	16S rRNA gene V4 region Platform: HiSeq (Illumina) Primers: 515F/806R QIIME2 Pipeline: Chimaera removal and sequence variants (ASVs) identification (Deblur) Taxonomic assignment: Greengenes database (2013) Normalisation: 1000 sequences per sample	Diversity and statistical analysis: Alpha-diversity: Shannon index Kruskal–Wallis rank sum test to evaluate the significance of mean values for each diversity calculation Beta diversity: Unweighted UniFrac and weighted UniFrac using relative abundances of ASVs PERMAONVA with Bonferroni correction to test marginal effects for organ type, sex, age, cause of death, PMI, and BMI
Ashe et al. (2021)	Handling: Samples transported on dry ice and stored at −80 °C Extraction: RNeasy PowerMicrobiome Kit (Qiagen)	16S rRNA gene V6-V8 region Platform: MiSeq (Illumina) Primers: B969F/BA1406R QIIME2 Pipeline: Quality control and denoised (Deblur) Taxonomic assignment (SILVA database) Normalisation: To the sample with the lowest total number of sequences. Additional sequencing: Metagenomic and Metatranscriptomic analyses	Diversity and statistical analysis: Alpha-diversity: Shannon index ANOVA to test significance by grouping samples by donor, ADD, and decomposition stage PCA at the phylum and genus level between sequencing methods
Liu et al. (2020)	Handling: Samples stored at −80 °C Extraction: QIAamp DNA Mini Kit (Qiagen) Controls: DNA extraction and PCR amplification included negative controls	16S rRNA gene V3 and V4 region Platform: IonS XL Primers: 341F/806R Mothur Pipeline: Quality Control and filter (Cutadapt) Chimaeras were filtered and trimmed (VSEARCH) OUT classification (UPARSE) Taxonomic assignment: SSUrRNA database in SILVA132 Taxonomic alignment (Greengenes reference (MUSCLE)) Normalisation: All samples set to the same number of reads, based on the sample with the lowest read count	Diversity and statistical analysis: Alpha diversity: Shannon, Chao1, Simpson and ACE indexes PCoA and NMDS to visualize the similarities or dissimilarities of variables Machine learning algorithms: Random forest Support vector machine Artificial neural network Validation: Internal validation by repeating the model 15 times
Deel et al. (2021)	Handling: Ribs individually bagged and frozen at −10 °C, transported on dry ice to Colorado State University and stored at −20 °C Extraction: Pulverised rib bone (0.2 to 0.5 g) used for DNA extraction, following a protocol that includes SDS (10%) for cell membrane lysis and Proteinase K for protein digestion. Controls: 15 extraction blanks included for controls	16S rRNA gene V4 region Platform: MiSeq (Illumina) Primers: Standard primers from the Earth Microbiome Project QIIME2 (2018.4) Pipeline: Quality filtered and denoised (Deblur) Taxonomic assignment (Greengenes 13_8 99%) Phylogenetic tree generation (SEPP) Normalised: 17,098 reads per sample	Diversity and statistical analysis: Alpha diversity: Faith’s phylogenetic diversity Beta diversity: Weighted and unweighted UniFrac distances PCoA for data visualization PERMANOVA to test effect sizes between season, hosts, and the first and last ADDs Machine learning algorithms Random forest
Zhang et al. (2021)	Handling: Samples were kept at −20 °C until further analysis Extraction: PureLink Genomic DNA Mini Kit (Invitrogen, USA)	16S rRNA gene V3 and V3-4 regions Platform: MiSeq (Illumina) Primers: 515F/806R QIIME Pipeline: Sequences clustered (UCLUST) Chimaera identification and removal (ChimeraSlayer) Taxonomy assignment: RDP classifier Identification: BLAST against Greengenes (2013) 97% reference Taxonomy alignment (PyNAST) Normalisation: 1000 sequences per sample	Machine learning algorithms: Metadata and taxonomic data merged Random forest xgboost method, Neural network Validation: 5-fold cross-validation
Zhao et al. (2022)	Handling: Samples were kept at −20 °C until further analysis, and extractions were stored at −80 °C Extraction: DNA Mini Kit (Qiagen)	16S rRNA gene V3-V4 regions Platform: Ion S5 XL platform Primers: 341F/806R Mothur Pipeline: Filter and trim reads (Cutadapt V1.9.1) Chimaera removal (UCHIME) Assigned to OTUs (UPARSE) Taxonomic assignment (Silva (v132) database)	Diversity and statistical analysis: Alpha diversity: Chao1, abundance-based coverage estimator, Shannon and Simpson indexes Beta diversity ANOVA to test variance in alpha diversity and beta diversity between groups Machine learning algorithms Random forest Validation: Cross-validation for feature screening
Iancu et al. (2023)	Handling: Samples stored at −20 °C until further analysis Shipped on ice to the University of North Dakota, USA Extraction: Blood and Tissue modified protocol (Qiagen)	16S rRNA gene V3-V4 regions, as well as metagenomics analysis (METAGENassist) Platform: MiSeq (Illumina) Primers: 357F/806R QIIME2 (2019.7) Pipeline: Sequences filtered (q2-demux) Denoising, phiX chimaera removal, and identification of ASVs (DADA2) Phylogeny tree generation (FastTree) ASV classification (SILVA SSU) Normalisation: 49,578 reads per sample	Diversity and statistical analysis: Alpha diversity and beta diversity analyses: Shannon’s diversity index and Bray-Curtis index Kruskal-Wallis pair-wise test to test the alpha group of significance and the difference between groups PCoA for visualisation PERMANOVA to determine distances between groups Validation: Cross-validation in the metagenomics analysis
Burcham et al. (2024)	Handling: Samples stored at −20 °C until further analysis. All samples were shipped to CU Boulder or Colorado State University on dry ice and stored at −20 °C Extraction: PowerSoil DNA isolation kit 96-htp (MoBio Laboratories) Controls: DNA extraction negative and no-template PCR control samples	16S rRNA gene V3-V4 regions, as well as metabolite extraction and Shotgun metagenomic sequencing Platform: MiSeq (Illumina) Primers: 515F/806RB QIIME2 Pipeline: Taxonomic assignment (SILVA 132 99%) Phylogenetic tree generation (SEPP method)	Diversity and statistical analysis: Alpha diversity: SV richness and Faith’s phylogenetic diversity formulas. Beta diversity: Generalised UniFrac method to calculate dissimilarity PERMANOVA for statistical comparisons Machine learning algorithms Random forestBatch Effect: Samples were randomly assigned to runs to negate batch effects. Validation: Internal validation and on an independent test set and nested cross-validation
Iancu et al. (2024)	Handling: Samples stored at −20 °C until further analysis Extraction: Blood and Tissue modified protocol (Qiagen)	16S rRNA gene V3-V4 regions Platform: MiSeq (Illumina) Primers: 341F/805R QIIME2 (v0.99.6) Pipeline: Denoising, chimaera removal (DADA2 (v.1.26.0)) Taxonomic assignment (SILVA 132 99%) Normalised: 46191.9 mean reads per sample	Diversity and statistical analysis: Alpha diversity: Between different individuals, locations, and snow ANOVA to test for significance Machine learning algorithms Random forest

**Figure 2:**
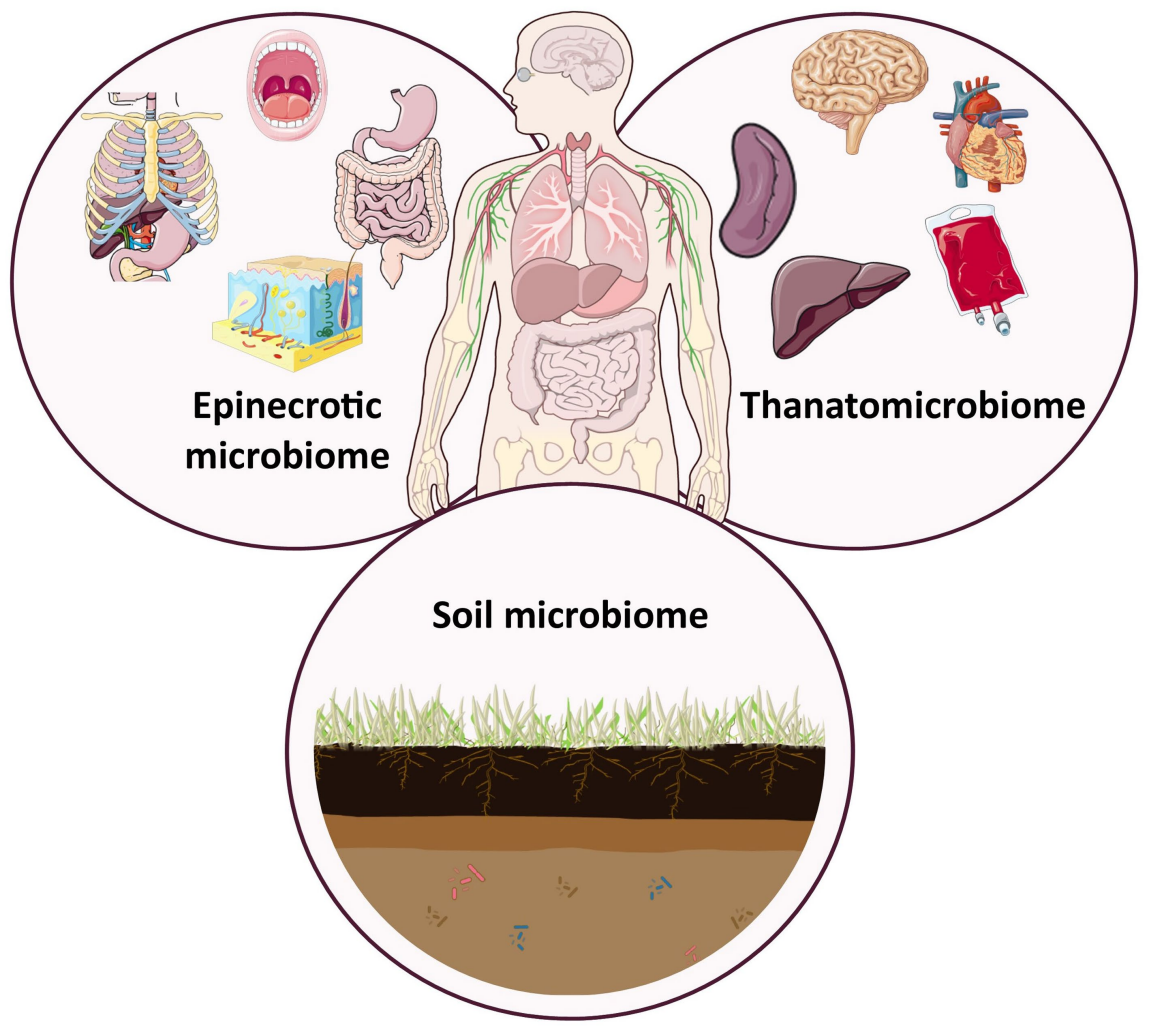
Intersection of the post-mortem microbiome, including the epinecrobiome, thanatomicrobiome and soil microbiome of mammalian remains. After Javan et al. (2016a, 2016b) and Wu et al. (2024). The artwork used in this figure was adapted from Servier Medical Art (https://smart.servier.com/). Servier Medical Art by Servier is licensed under a Creative Commons Attribution 3.0 Unported License.

The characterisation of the mammalian post-mortem microbiome, including the thanatomicrobiome (consisting of microbial communities found internally in blood, organs and fluids) (Javan et al., 2016b), the epinecrotic microbiome (consisting of microbial communities found externally on the surfaces of the body, with their roles elucidated in the decomposition of mammals) (Pechal et al., 2014), and soil microbiome (Olakanye et al., 2014; Procopio et al., 2019) have provided forensic scientists with a tool to aid in time-since-interval estimation (Figure 2). The use of microbial communities to aid in PMI estimation has become more prominent since proposing their application as a post-mortem microbial clock (Metcalf et al., 2013). Changes in the form of shifts in the abundance (how many) and diversity (variety and type) of microbial communities coincide with the physiochemical changes of the body as decomposition progresses. Understanding the shifts (when microbial communities appear, proliferate, and decline) over time, for different burial and environmental conditions, provides a microbial timeline that can act as a clock, allowing forensic scientist to estimate the PMI of a body (Metcalf et al., 2013; Finley et al., 2015).

Several reviews have been undertaken to elucidate the role of microbial communities within forensic investigations, specifically to present an overview of how microbial communities can be adopted for PMI estimation (Metcalf, 2019; Jangid et al., 2023; Moitas et al., 2023), the succession of the thanatomicrobiome (Javan et al., 2019; Zapico and Adserias-Garriga, 2022) and the epinecrotic microbiome (Dash and Das, 2020), and their use the PMI estimation of advanced decomposition (Franceschetti et al., 2023), as well as to present a comparison of microbial fingerprinting techniques in forensic science in estimating the PMI (Finley et al., 2015). Yet, none of the reviews consider the role of gravesoil microbial communities for utilisation beyond PMI estimation to grave location. Consequently, within the reviews, and the broader body of knowledge, there is a lack of clarity, which has led to confusion, especially when describing PMI and PBI for buried remains (Forbes, 2008), where these two time-since-intervals are sometimes used interchangeably (Procopio et al., 2019; Zhang et al., 2021). For application in criminal investigations and legal proceedings it is imperative that a clear distinction on the use, meaning and purpose of these post-mortem intervals are made. This will ensure that appropriate methods are developed to provide crucial and precise information related to the victims remains. Solving crime and aiding in victim identification is at the centre of forensic research, but it requires targeted approaches that are reliable and robust.

Forensic ecogenomics (Ralebitso-Senior, 2018), a sub-discipline of forensic microbiology (Carter et al., 2017), can aid clandestine grave identification through the use of molecular microbial ecology approaches to analyse gravesoils. Since less than 1% of bacterial communities can be cultured (Amann et al., 1995), microecophysiology approaches such as denaturing gradient gel electrophoresis (DGGE) (Muyzer et al., 1993; Zhang and Fang, 2000; Olakanye et al., 2014, 2015; Iancu et al., 2015), polymerase chain reaction (PCR), and terminal restriction fragment length polymorphisms (T-RFLP) (Parkinson et al., 2009; Handke et al., 2017), are useful tools to measure and characterise shifts in microbial communities from gravesoil during decomposition (Ralebitso-Senior et al., 2016). The ability of DGGE and T-RFLP methods to characterise microbial communities from soils is useful due to their low cost and easy data analysis, which makes them favourable for quick analysis and in cases where forensic laboratories do not have access to massively parallel sequencing (MPS) (Lerner et al., 2006; Thies, 2007; Jousset et al., 2010; Lenz and Foran, 2010; Bergmann et al., 2014; Jurkevitch and Pasternak, 2021; Olakanye and Ralebitso-Senior, 2022). However, these techniques are limited by the resolution and depth of taxonomic data (Lerner et al., 2006; Handke et al., 2017). Forensic ecogenomic approaches paired with MPS can be used to locate clandestine burials through the analysis of shifts within gravesoil microbial communities. Considering the use of microbial communities in PMI estimation, we are positing their use in PBI as well as in a new concept called the time-since-translocation [post-translocation interval (PTI)]. It is argued that integrating forensic ecogenomic approaches, which uses molecular microbial ecology techniques to analyse changes to ecosystems as a result of the decomposition process, could enhance the accuracy of PBI and PTI estimations (Ralebitso-Senior, 2018). The conceptual framework within this review addresses this by discussing the PBI and PTI using gravesoil microbial communities, and showing, for the first time, their relationship with the PMI. The framework and synthesis of knowledge is based on a broad review of literature focused on the application of the post-mortem microbiome in forensic science. The literature search was conducted between October 2024 and January 2025 using the Web of Science and Scopus databases and the Google Scholar academic search engine. Searches were conducted using the keywords “microbiome,” “post-mortem interval,” “time since death,” “post-burial interval,” “time since burial,” “soil microbiome,” “relocation,” and “exhumation.” Additionally, reference lists from reviewed articles were also scanned for additional citations. This search strategy was employed because it affords flexibility in the exploration of concepts and the mapping of emerging research themes and approaches within the literature. Given the growing research activity that emphasises the role of microbial communities in decomposition, this review provides more details for the use of forensic ecogenomics. The focus will be on time-since-interval estimation for terrestrial environments and will not include a discussion on the estimation of the post-mortem submersion interval (PMSI) for aquatic environments. In-depth discussions regarding the PMSI have been conducted previously (Dickson et al., 2011; Humphreys et al., 2013; Benbow et al., 2015; Cartozzo et al., 2021).

## 2 The three time-since-intervals

The three time-since-intervals (PMI, PBI and PTI) offer insight regarding the circumstances surrounding the disappearance and death of an individual. Their purpose also extends to narrowing the investigation window, revealing information regarding the manner and time of death (whether it was accidental or intentional), assisting in estimating the time gap between death, body movement and disposal (or the perimortem and post-mortem behaviour of perpetrator) and potentially connecting suspects to crime scenes at specific points in time (Turner and Wiltshire, 1999; Introna et al., 2011; Sharma et al., 2015). The main function of the PMI estimation within forensics is used to aid in victim identification. Its estimation is based on either the observable physiological changes of the body, or from the thanatomicrobiome and the epinecrotic microbiome of the body (Can et al., 2014; Pechal et al., 2014, 2018; Damann et al., 2015; Hauther et al., 2015; Iancu et al., 2015, 2016, 2023, 2024; Javan et al., 2016b, 2017; Johnson et al., 2016; DeBruyn and Hauther, 2017; Liu et al., 2020; Lutz et al., 2020; Ashe et al., 2021; Deel et al., 2021; Zhang et al., 2021; Zhao et al., 2022; Burcham et al., 2024), to provide a timeframe for the period that has passed since death. This information is useful because it can help to identify individuals, generate investigative leads by following up on their behaviour prior to the crime (from CCTV footage, for example), and narrow a suspect pool (Simmons, 2017).

**Figure 3:**
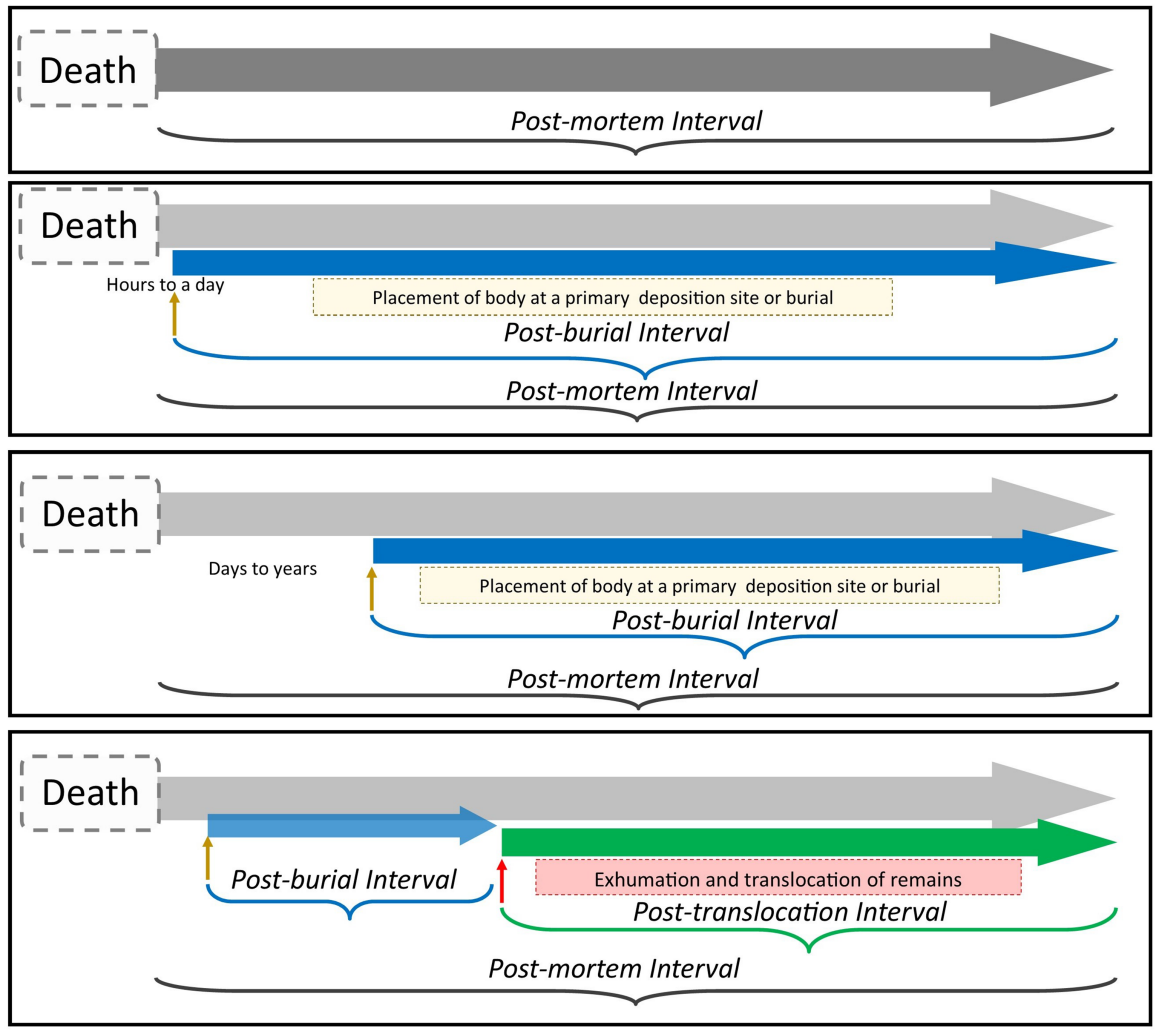
Schematic showing the ordering and overlap of the post-mortem timelines at a primary deposition or burial site. The PMI is inclusive of the PBI and the PTI.

The time-since-burial (PBI) and the time-since-translocation (PTI), while distinct in their function, form two parts that can be used to infer information about the broader time-since-death (PMI) (Figure 3). The PBI and PTI, while contributing to victim identification, are primarily focussed on providing a contextual interpretation and temporal estimation of the treatment of the body in relation to the grave and deposition site. Here, the burial environment and specifically the grave soil becomes the focus. There are some caveats that need to be considered regarding the PBI and PTI. In cases concerning buried remains, the PBI and PMI can refer to the same time-since-interval or to two separate events. The first instance occurs when a body is placed in a surface deposition environment or a grave within the first couple of hours to a day after death (Burcham et al., 2021). In this scenario, the PMI and the PBI, within a margin of error, can be used to calculate the time-since-death (Forbes, 2008; Singh et al., 2016). However, this information is rarely available at the time a body is discovered in forensic cases. Perpetrators might try to conceal and destroy the evidence of the crime and prevent victim identification to avoid being caught (Kamaluddin et al., 2018). To avoid detection by police, perpetrators can delay depositing the victim’s remains in a burial environment immediately after death (Byard, 2024; Ploeg et al., 2024). Reasons for delaying depositing or burying the remains could include that the offender wanted to conceal the remains to avoid suspicion or confuse the investigation, which delays the likelihood of an arrest or conviction (Tumer et al., 2013; De Matteis et al., 2021; Byard, 2024). Since deposition or burial might have taken place sometime after death, the estimation of the PBI should be considered distinct from the PMI. In which case the PBI will be shorter than the PMI (Forbes, 2008; Watson et al., 2021).

The movement of the body from its original deposition or burial site can complicate the interpretation of the crime scene and the PMI estimation. Post-mortem translocation of a body from its original burial or deposition site can occur due to several reasons, such as the post-mortem movement or contractions after death, shifting a body from its original location (Wilson et al., 2020), religious or cultural exhumation or reburial, as has been observed in the archaeological record (Weiss-Krejci, 2005; Carroll, 2009), natural disasters (Magni and Guareschi, 2021) or through scavenger activity (Haglund et al., 1989; Berryman, 2002; Young, 2017). Bodies are also moved as part of legal case work (Morovic-Budak, 1965), or as part of ongoing humanitarian work related to conflict, as exemplified by Ferrándiz (2013) for mass graves in Spain. Post-mortem translocation can also occur within forensic contexts. After a victim has been murdered, perpetrators, depending on their relationship with the victim, the context and the location of the crime, might use a range of body disposal methods as their *modus operandi* (Beauregard and Field, 2008; Chai et al., 2021; Terribile et al., 2024; Whitehead Apm et al., 2024). One such approach could be to hide the body or move it from the primary crime scene or hiding place to a shallow grave (Davenport, 2001; Hawksworth and Wiltshire, 2011; Berezowski et al., 2022). To distinguish this post-mortem movement of the body from the time-since-death (PMI) and time-since-burial (PBI), we define the period since remains were intentionally removed and relocated by perpetrators as the post-translocation interval (PTI). The estimation of the PTI as a post-mortem clock can be used in cases involving the intentional post-mortem movement of buried remains to a secondary locale away from the original crime scene in forensic investigations. Within the broader time-since-death interval, the PTI occurs after body deposition or burial at a primary locale. Once the remains are moved to a secondary (or sometimes a tertiary) location the secondary PBI (or tertiary PBI) commences. While PMI and PBI, depending on the context, can refer to one or two separate post-mortem events, the PTI will be the period in the post-mortem timeline, after the PBI, when a body is excavated and translocated to a secondary locale. There have been limited studies investigating the post-mortem movement of remains by perpetrators. The limited number of reported or published cases in which perpetrators have removed remains from the primary locales and translocated them to secondary locales could potentially explain why relatively little attention has been directed to PTI estimations. Notwithstanding this, cases of translocated remains have been reported, as exemplified by incidents where the remains of victims were moved by perpetrators in Bosnia and Serbia (Skinner et al., 2001; Jessee and Skinner, 2005; Congram, 2008; Tuller and Hofmeister, 2014; Klinkner, 2016), and more recently in the United States (Shapiro, 2020). The estimation of the PBI and PTI contributes to investigations as the intervals may be useful to link a suspect to a crime scene, especially in cases where there is no reliable witness testimony or other circumstantial evidence. Collectively, this information is needed by police for crime reconstructions to address key questions of who, what, why, when and how; and crucially by prosecutors to make sound judgments in court proceedings, avoiding wrongful prosecutions (Introna et al., 2011).

## 3 The intersection between microbial communities, forensic ecogenomics and post-mortem time-since-interval estimations

### 3.1 Microbial activity during decomposition: the post-mortem interval

**Figure 4:**
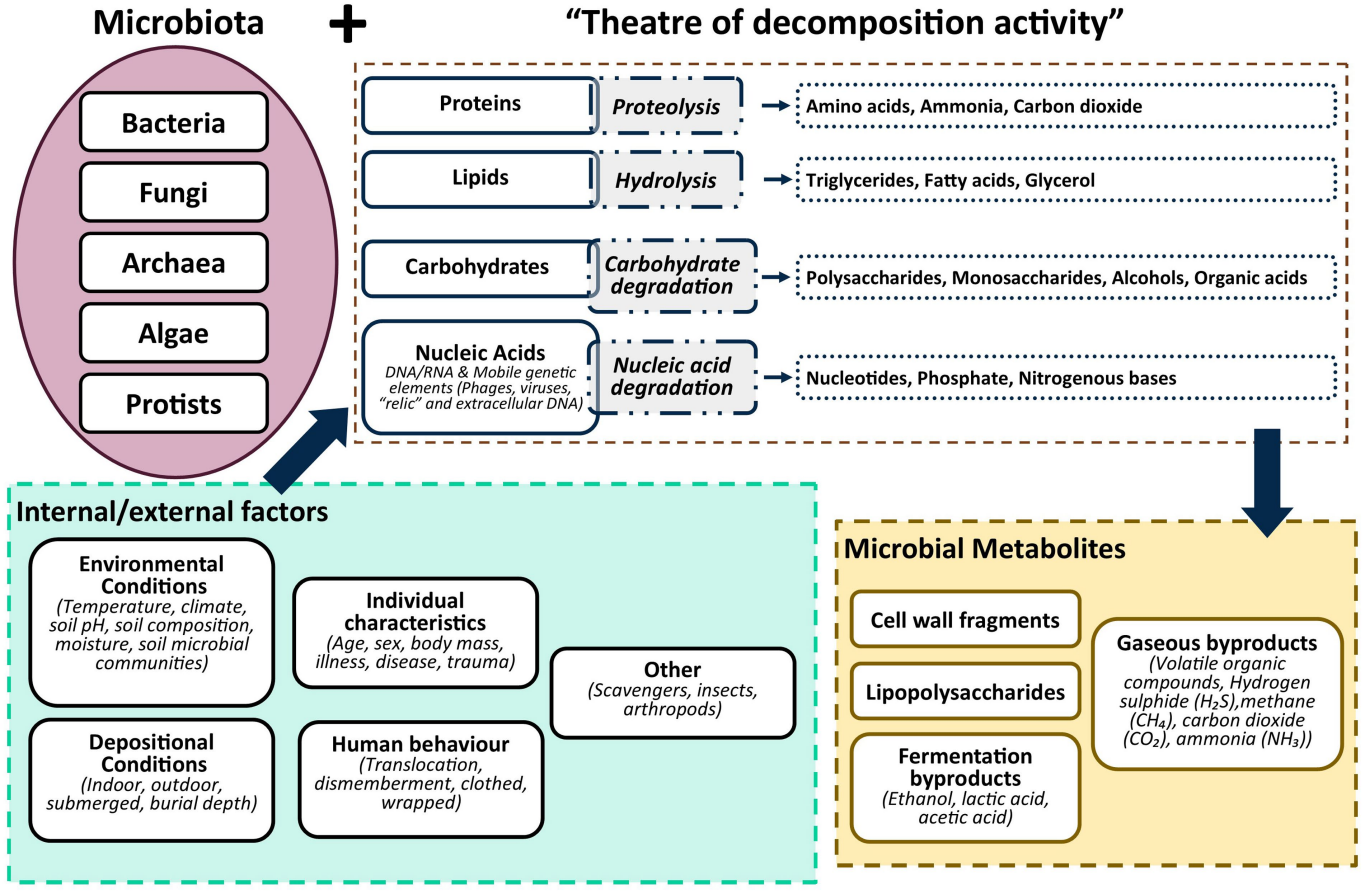
Theatre of decomposition activity based on the microbial “theatre of activity” modelled after Whipps et al. (1988) and Berg et al. (2020).

The microbiome of individual human beings is unique and consists of a diversity and abundance of bacteria, archaea, fungi, and algae across different regions of the body (Huttenhower et al., 2012), where they vary depending on their “theatre of activity” (Whipps et al., 1988; Berg et al., 2020). The “theatre of activity” encompasses the collective genetic material of the microbes, the products of their metabolic activities, and molecules produced in the environment in which they function and interact (Whipps et al., 1988; Berg et al., 2020). Microbial communities like bacteria, that live on skin and within the digestive system, genital tract, and oral cavity of mammals, play a crucial role in maintaining immune systems, protecting against pathogens and breaking down and metabolising complex molecules (Jordán et al., 2015; Cundell, 2018; Rowland et al., 2018; Lambring et al., 2019; Moeller and Sanders, 2020). In death, these microbial communities play an equally central role during decomposition, taking centre stage in what we designate as the ‘theatre of decomposition activity’ (Figure 4). During decomposition of the body, they are crucial in the biochemical breakdown of structural elements (Janaway, 1995; Gill-King, 1996; Hopkins et al., 2000; Parkinson et al., 2009) as complex molecules like proteins, lipids, carbohydrates, and nucleic acids are broken down into simple molecules (Mackie et al., 1991; Vass et al., 2002; Hyde et al., 2013; Fiedler et al., 2015; Forbes et al., 2017; Lutz et al., 2020; Nolan et al., 2020; Burcham et al., 2024). During the biochemical breakdown, microbial metabolites, or the byproducts of the decomposition process, are released (Agapiou et al., 2015; Irish et al., 2019; Furuta et al., 2024). A combination of biotic and abiotic factors can alter, impede or accelerate the decomposition process and the biochemical breakdown of the structural elements (Carter, 2005; Schotsmans et al., 2017, 2022; Young, 2017; Spies et al., 2020, 2024). Within terrestrial ecosystems decomposer communities such as bacteria and fungi have evolved to take advantage of decaying organic matter (DeBruyn et al., 2024). The sensitivity and transiency of microbial communities in response to the pulse of nutrients that are released into the soil, creating a Cadaver Decomposition Island (CDI) (Carter, 2005), make them a valuable indicator of internment and exhumation of bodies (Humphreys et al., 2013; Pechal et al., 2013; Benbow et al., 2015; Cobaugh et al., 2015; Hyde et al., 2017). This is especially the case considering the impact of decomposition and decay on the soil biogeochemistry and microbial community composition (Hopkins et al., 2000).

Temporal and compositional shifts in the post-mortem mammalian microbiome (thanatomicrobiome, and the epinecrotic), have facilitated the estimation of the PMI (Metcalf et al., 2013; Bergmann et al., 2014; Can et al., 2014; Olakanye et al., 2014; Pechal et al., 2014; Javan et al., 2016b, 2016a, 2017). The analysis of microbial community composition through MPS has revolutionised forensic researchers’ ability to rapidly characterise diverse microbial communities from the soil microbiome, the thanatomicrobiome, and the epinecrotic microbiome, thereby enhancing forensic analyses (Table 1). MPS is becoming the preferred method to sequence the hypervariable V3-4 region of the 16S rRNA (Gao et al., 2021) because of its improved capacity to characterise diverse microbial communities rapidly from the body (gut, oral, anal cavities and skin and organs) (Hyde et al., 2013; Pechal et al., 2013, 2014; Iancu et al., 2016, 2024; Javan et al., 2016a; Burcham et al., 2024) and gravesoil (Finley et al., 2016; Cui et al., 2022; Olakanye and Ralebitso-Senior, 2022). Complex computational microbial community analysis is achieved through a bioinformatics pipeline [QIIME (Caporaso et al., 2010), QIIME2 (Bolyen et al., 2019), and Mothur (Schloss et al., 2009)] (Table 2). These pipelines allow for the characterisation of raw sequence data into Operational Taxonomic Units (OTU), with a cutoff at 97%, or more recently into Amplicon Sequence Variants (ASV), with a cutoff at 99%, due to the need for higher taxonomic resolution (Gao et al., 2021; Fasolo et al., 2024). Taxonomic assignment is achieved through reference databases, such as the Ribosomal Database Project (RDP) (Wang et al., 2007) which is useful for genus-level assignments or with SILVA (Quast et al., 2013) and Greengenes (DeSantis et al., 2006) which are databases preferred for species-level classification, and visualisation options (phylogenetic tree generation) (Liu et al., 2024).

**Table 1:** Study matrix summarising experimental models, environmental conditions, sequencing approaches, and key findings in reviewed studies estimating the PMI from microbial communities.

Study	Characteristics of the burial environment	Sampling location and days	Key taxa shifts	Findings and accuracy
Can et al. (2014) Human donor cadaver (*n* = 11) PMI: 20–240 h	Environment: Alabama Department of Forensic Sciences Medical Laboratory’s morgue	Sample: Dissection of organs with a sterile scalpel into sterile bags; blood was drawn from the heart with sterile syringes Controls: – Sampling time: Single samples from blood and internal organs (brain, heart, liver, spleen)	*Clostridium sordellii, Clostridium difficile, Clostridium bartlettii, Clostridium bifermentans and Clostridium limosum* are dominant in shorter PMI *Clostridium haemolyticum, Clostridium botulinum*, and *Clostridium novyi*, as well as *Escherichia coli* and *Escherichia albertii* are dominant in longer PMI	Thanatomicrobiome signatures are similar within the same individual cadaver’s organs, but differ across the cadavers, likely due to different PMIs
Lauber et al. (2014) Mice (*n* = 80) PMI: 48 days	Deposition condition: Placed on its right side on top of the soil Environment: University of Colorado. Juget series and classified as sandy-skeletal, mixed, frigid lithic Haplustolls	Sample: Sample with sterile swabs the skin on the head, belly, and the abdominal cavity, as well as gravesoil Control: – Sampling time: Day 0, 3, 6, 9, 13, 20, 34, 48	Untreated soil: M*organella* and *Proteus* increased abundance in late decomposition Sterilized soil: *Burkholderia*, *Novosphingobium*, *Staphylococcus*, and *Stenotrophomonas* were more abundant during active and advanced decay. *Bacillus* spp. higher in abundance during active and advanced decay	Sterilized soils slow down decomposition
Pechal et al. (2014) Pigs (*n* = 3) PMI: 7 days	Deposition conditions: Randomly placed on surface 20 m apart, covered with anti-scavenging cages Environment: Midwestern temperate forest habitat, Xenia, OH, USA	Sample: Sampling by swabbing of the buccal cavity and the skin with sterile cotton applicators Control: – Sampling time: Days 0, 1, 3, and 5	Phylum: Proteobacteria was the dominant followed by Firmicutes Proteobacteria decreased over the 5 days. Firmicutes became the dominant as decomposition progressed Family: *Moraxellaceae* was the most dominant, followed by *Pasteurellaceae, Enterobacteriaceae,* and *Aerococcaceae.* On the fifth day *Planococcaceae* was dominant, followed by *Clostridiales incertae sedis XI* and *Clostridiaceae*	Bacterial communities estimated PMI within 2–3 h after death (up to 94.4% with specific family-level taxa)
Damann et al. (2015) Human donor cadaver (*n* = 12) PMI: 27–1,692 days	Deposition conditions: Bodies placed on the ground surface Environment: Anthropological Research Facility, Knoxville, USA. Deciduous forest biome. Coghill–Corryton soil complex	Sample: Single lower rib from each cadaver Control: Soil samples from 1 km south of the facility Sampling time: One sampling point (when body decomposed enough to facilitate rib collection)	Phylum: Proteobacteria were the most dominant phyla across all four-sample group, followed by Firmicutes and Bacteroidetes. Actinobacteria and Acidobacteria were more dominant in the dry remains and soil samples than in the first two decay stages. For groups A, B, and C, Alphaproteobacteria increased. While Gammaproteobacteria decreased. Family: Bone samples: *Pseudomonadaceae, Clostridiaceae, Tissierellaceae, Caulobacteracea,* and *Sphingobacteriaceae,* Soil samples: *Hyphomicrobiaceae, Koribacteraceae, Solibacteraceae,* and *Flavobacteriaceae*	Differences in microbial composition can be observed between partially skeletonised and fully skeletonised dry remains
Hauther et al. (2015) Human donor cadavers (*n* = 12); 6 bodies were controls PMI: 20 days	Deposition conditions: Placed on site unclothed and uncovered Environment: Anthropological Research Facility, Knoxville, USA. Deciduous forest biome. Coghill–Corryton soil complex	Sample: Swabbing of cecum through an incision covered with duct tape. Six individuals were sampled daily Controls: Six cadavers were used as controls and were sampled only once Sample time: Individual sampling for the six bodies were at: 9 days (205 cumulative degree hours (CDH)), 14 days (408 CDH), 14 days (313 CDH), 15 days (330 CDH), 20 days (478 CDH), and 20 days (595 CDH) Control samples were collected at 0, 0, 44, 224, 230, and 330 CDH	*Bacteroides* and *Lactobacillus* declined in all individuals	Individual variability was noted; these differences could not be explained by cause of death, sex, or weight *Bacteroides* and *Lactobacillus* are reliable indicators for PMI
Javan et al. (2016a) Human donor cadaver (*n* = 27) PMI: 3.5–240 h	Study condition: Alabama and Florida morgues Environment: Indoor	Sample: Samples derived from blood, brain, buccal cavity, heart, liver, and spleen. Swab samples were collected using a sterile cotton tip CultureSwab applicator (buccal cavities and blood). Sections of the internal organs were dissected using sterile scalpels and placed in polyethylene bags. Blood samples (heart and femoral veins) were placed in 10 mL BD vacutainer EDTA tubes Control: –Sampling time: Single sampling time in morgue	Most abundant order in females Clostridiales, and the most abundant genus in females was *Pseudomona*s. The most abundant taxa in the mouth were *Clostridium, Clostridiales*, and *Streptococcus* and *Rothia* Taxa changed in abundance over time across sexes and sample types*, unknown Clostridium* sp.*, Clostridium novyi, Prevotella bivia* and *Prevotella timonensis.* *C. novyi* was more abundant during late PMI; while *unknown Clostridium species* was more abundant during early decomposition	Specific organ dependant changes in microbial composition were observed during the decomposition Additionally, *Clostridium* spp. appears to be a key biomarker of PMI estimation in human cadavers
Johnson et al. (2016) Human donor cadaver (*n* = 21) PMI: 500 accumulated degree days (ADD)	Deposition conditions: Bodies placed unclothed on the ground in a prostrate position Environment: Anthropological Research Facility, University of Tennessee at Knoxville, USA. Temperate deciduous forest, well-drained fine textured clayey soils	Sample: The mose and ear canal was swabbed which was then placed in a collection tube, wrapped in a sterile collection bag Control: – Sampling time: Single collection per cadaver. An exception is 4 cadavers were swabbed continuously for 2–3 days after placement	The phyla Actinobacteria and Armatimonadetes were most predicative, followed by Planctomycetes, Verrucomicrobia, and Cyanobacteria	Genus and family are more informative for the development of a predictive models for PMI estimation. In the ear samples, microbial diversity decreased as PMI progressed PMI of unknown samples: MAE of ±55 ADD
DeBruyn and Hauther (2017) Human donor cadaver (*n* = 4), Controls (*n* = 6) PMI: 30 days	Deposition conditions: Surface placement Environment: Anthropological Research Facility University of Tennessee at Knoxville, USA. Temperate deciduous forest, well-drained fine-textured clayey soils Summer season	Sample: Swabbing of the cecum through a small incision that was covered with tape Control: Swabbing of the cecum through a small incision Sampling time: Re-sampled daily until remains were in too advanced decomposition, control samples were only collected once	Early Communities: *Bacteroides* and *Parabacteroides* (Phylum: Bacteroidetes), and the Firmicutes *Faecalibacterium, Phascolarctobacterium, Blautia*, *Lachnospiraceae incertae sedis.* Late Communities: Clostridiales within phylum Firmicutes (*Clostridium, Peptostreptococcus,* and *Anaerosphaera*), and Gammaproteobacteria (*Wohlfahrtiimonas, Ignatzschineria, Acinetobacter and Providencia*)	Decreased diversity as decomposition progressed Generally, there was a decline in Bacteroides and an increase in Clostridium
Javan et al. (2017) Human donor cadaver (*n* = 45) PMI: 4–78 h	Study condition: Alabama Department of Forensic Sciences in Montgomery and the Office of the District One Medical Examiner in Pensacola, USA Environment: Indoor	Sample: 10 mg of liver and spleen tissues were dissected using sterile scalpels and placed in polyethylene bags Control: – Sampling time: Single collection per cadaver	Clostridium spp. dominant in majority (95%) of samples (liver and spleen), and detected in the early post-mortem period	Forensically relevant bacteria identified in the V3 region compared to the V3-4 region
Pechal et al. (2018) Human cadavers from routine death investigations (*n* = 188) PMI: 24–48 h	Deposition conditions: Case-specific natural deaths, accidental deaths, suicides and homicides Environment: Collected at Wayne County Medical Examiner’s Office, Michigan, USA	Sample: Swabs with DNA-Free sterile cotton-tipped applicators from the ear, eyes, nose, mouth, umbilicus, and rectum Control: – Sampling time: Single collection point	24,25–48 h: High abundance of *Streptococcu*s in eyes, while *Haemophilus parainfluenzae* and *Streptococcus* were more abundant in the mouth Mouth: Dominant taxa in the 24 h: *Streptococcus, Haemophilus, Veillonella*; anaerobic genera (*Prevotella, Fusobacterium*), and *Rothia* For all anatomical areas, at phylum level Actinobacteria and Bacteroidetes decreased in abundance after 2 days post-mortem, while Proteobacteria abundance increased. Firmicutes (*Staphylococcus* and *Streptococcus*) also decrease after 2 days, expect for the nose	Post-mortem microbiome diversity changes over time and between anatomical sites
Lutz et al. (2020) Human cadavers from criminal casework (*n* = 40) PMI: 24–432 h	Deposition conditions: Case-specific natural deaths, accidental deaths, suicides and homicides Environment: Department of Public Health, Experimental and Forensic Medicine Morgue, University of Pavia in Pavia, Italy	Sample: Tissue samples (brain, heart, liver, spleen, prostate, and uterus). Tissue samples were collected and placed into labelled sterile polyethylene bags Control: – Sampling time: Single collection point	Clostridiales and the family Saprospiraceae were the most dominant taxa identified form internal organs. Burkholderiales (heart) and Clostridiales (all organs except uterus) increased as decomposition progressed, while taxa from order MLE1-12 decreased (brain, heart, liver, and spleen) as decomposition progressed	Individual characteristics (sex, age, cause of death, PMI, and BMI) affect microbial diversity Microbial succession in internal organs can be used to estimate PMI
Ashe et al. (2021) Human donor cadavers (*n* = 3) PMI: 253–392 ADD	Deposition conditions: Placed on the ground in a supine position Environment: Forensic Osteology Research Station, Western Carolina University, Cullowhee, USA. Southern Appalachian Mountains Spring season	Sample: Oral samples collected using sterile swabs, placed in a microcentrifuge tube Control: – Sampling time: 5–7 times based on decomposition	Firmicutes dominated the early and middle sampling times, followed by Proteobacteria and then Actinobacteria. R*othia* spp. and *Lactobacillus* spp. found in early decomposition, while in middle to late decomposition common taxa were Streptococcus spp., *Bacillales* spp., and *Planococcaceae* spp. *Pseudomonas* spp. present in later decomposition	Temperature differences at the deposition site affected the microbial communities Microbial shifts could be observed at Phylum level, but the Genus level provided better resolution of PMI estimation from the oral microbiome
Liu et al. (2020) Mice (*n* = 240) PMI: 15 days	Deposition conditions: Placed on sterile plates with ambient temperature and moderate relative humidity Environment: Experimental Animal Centre of Xi’an Jiaotong University, China	Sample: Internal organs (brains, hearts and ceca) Control: – Sampling time: 0 h, 8 h, 12 h, 1 day, 2 days, 4 days, 7 days, 10 days, 13 days and 15 days	Brain: Family level *Enterobacteriaceae* and *Peptostreptococcaceae,* Heart: Family level *Enterobacteriaceae* and *Lactobacillaceae,* The heart and brain: Genus *Morganella and Proteus.* At the species level *Clostridium novyi, Proteusvulgaris, Anaerosalibacter bizertensis,* and *clostridium butyricum* Cecum: Family level *Lactobacillaceae, Enterococcaceae* and *Erysipelotrichaceae.* At genus level, *Lactobacillus, Enterococcus and Dubosiella,* and at the species level*, Lactobacillus reuteri, Enterococcus faecalis* and *Firmicutes bacterium M10-2* Advanced decomposition in the brain, heart and cecum dominant taxa: *Enterococcus faecalis, Clostridium cochlearium* and *A. bizertensis*	As decomposition progressed, the microbial communities of the brain, heart, and cecum became more similar MAE of 1.5 ± 0.8 h within 24-h of decomposition and 14.5 ± 4.4 h within 15-day decomposition
Deel et al. (2021) Human donor cadaver (*n* = 6) PMI: 1–9 months	Deposition condition: Three bodies were placed in cages per season Environment: Southeast Texas Applied Forensic Science Facility, Huntsville, USA Spring and summer seasons	Sample: Right and left lower ribs (a total of 8 bones from each individual) Control: – Sampling time: ampled every 3 weeks	Dominated by Proteobacteria Fresh to advanced decay: G*ammaproteobacteria* and *Actinobacteria*, and taxa unclassified *Pseudomonadaceae, Pseudomonas, Acinetobacter*, and two different *Corynebacterium.* *Phyllobacteriaceae* and *Devosia* increased in abundance as decomposition progressed.	Bone decomposer microbiome is distinct from the skin microbiome and the soil microbiome in decomposition, this also varies by season Summer model: MAEs from 724 to 853 ADD over a total of 5,201 ADD, error of ± 39 days Summer and spring model: MAEs from 793.33, error of ± 34 days
Zhang et al. (2021) Human cadavers from routine death investigations (*n* = 188) (Data from the Pechal et al. (2018) study) PMI: 24–48 h	Deposition conditions: Case-specific natural deaths, accidental deaths, suicides and homicides Environment: Collected at Wayne County Medical Examiner’s Office, Michigan, USA	Sample: Swabs with DNA-Free sterile cotton-tipped applicators from the ear, eyes, nose, mouth, umbilicus, and rectum Control: – Sampling time: Single collection point	48 h PMI: Dominated by *Streptococcus* sp. PMI between 49 and 72 h: Dominated by *Moraxellaceae* 73 h or higher PMI: Increased *Veillonella dispar* sp. and *Proteus* sp.	Accuracy of methods: xgboost method highest accuracy (74.5–87.6%) Neural network (70.7–83.0%) Random forest (73.6–86.3%) Highest accuracy was achieved for xgboost when considering a combination of the five anatomic areas: ears, eyes, nose, mouth, and rectum (77.5%)
Zhao et al. (2022) Rats (*n* = 96) PMI: 59 days	Deposition conditions: Placed on room to decompose Environment: In an animal room at Nanjing Agricultural University, Nanjing, China	Sample: Swabs of oral cavity Control: – Sampling time: 0 h, day 1, day 3, day 5, day 10, day 15, day 20, day 24, day 30, day 40, day 52 and day 59	Phylum: Pre-rupture: *Proteobacteria dominant* After 20 days: Firmicutes are dominant Genus: 0 h: *Acinetobacter* dominant *Enterococcus*, *Bacteroides* and *Proteus* were dominant between Day 3 to 5, but decreased after 10 days Later PMI: *Ignatzschineria* and *Cerasibacillus* more abundant	Oral microbiome shifts during decomposition R^2^ = Model accuracy was 93.94% for PMI estimation
Iancu et al. (2023) Human donor cadaver (*n* = 8) PMI: 0–12 h	Deposition conditions: Bodies observed for 12 h in the morgue Environment: Institute of Legal Medicine Iasi, Romania	Sample: Samples of face and hands skin collected with sterile cotton swabs. Control: – Sampling time: Sampled twice over 12 h (0 h on arrival and 12 h later)	Phylum: Firmicutes and Actinobacteria higher at 0 h, as decomposition progressed, Proteobacteria and Bacteroidetes abundance increased Genus: *Staphylococcus and Peptoniphilus* are present at 0 h, but increase in *Streptococcus, Lactobacillus*, *Clostridium, Micrococcus* and *Enterobacter* after 12 h	Antemortem health and lifestyle conditions affect the post-mortem microbiome
Burcham et al. (2024) Human donor cadaver (*n* = 36) PMI: 21 days	Deposition conditions: Placed supine and unclothed on the soil surface Environment: Colorado Mesa University Forensic Investigation Research Station (FIRS), Sam Houston State University Southeast Texas Applied Forensic Science (STAFS) Facility and University of Tennessee Anthropology Research Facility (ARF) Spring, summer, fall and winter seasons	Sample: Sample of skin surface (head), torso (hip) and gravesoils with a sterile swab Control: Control soil samples Sampling time: 21 days	Key microbial decomposers: identified: *O. alkaliphila*, *Ignatzschineria*, *Wohlfahrtiimonas*, *Bacteroides*, *Vagococcus lutrae, Savagea*, *Acinetobacter rudis* and *Peptoniphilaceae*	Inter-domain microbial decomposers found on cadavers during decomposition Estimate PMI within ±3 days
Iancu et al. (2024) Pigs (*n* = 3) PMI: 23 weeks	Deposition conditions: Placed on the ground at a distance of 20 m between each covered with a wire cage Environment: Mekinock Field Station, University of North Dakota, Grand Forks, North Dakota, USA. Located between agricultural fields (corn, soybeans, and wheat crops). Characterized by tallgrass prairie. Field covered with snow (depth up to 130 cm) Winter season	Sample: Triplicate tissue swabs from the nose (externally and internally) Control: – Sampling time: Samples collected weekly for 23 weeks	Phylum Firmicutes (*Clostridia and Bacilli*) dominated weeks 1–7, followed by Proteobacteria (mainly *Gammaproteobacteria*) and Actinobacteriota (mainly A*ctinobacteria*). Proteobacterial becomes dominant at week 8. From week 12–16 Proteobacterial and Firmicutes have relatively similar abundance. Week 17–23 Protrerobacteria dominant. Week 23 an increase in Campylobacterota (Campylobacteria) and Bacteroidota Genus: Psychrobacter increased from week 5–10, Pseudomonas increased weeks 5–9 and week 18. Moraxella abundance decreased after week 5, Clostridium abundance fluctuates, high abundance from weeks 1–7, decreases by weeks 8–10 and increased weeks 11–16	Best model based on internal and external swabs: MAE of 1.36 weeks Accurate PMI of 9.52 days in severe cold weather

To evaluate the reliability of the post-mortem microbiome, a diverse range of models [human donors (Can et al., 2014; Damann et al., 2015; Hauther et al., 2015; Johnson et al., 2016; DeBruyn and Hauther, 2017; Javan et al., 2017; Ashe et al., 2021; Deel et al., 2021; Iancu et al., 2023; Burcham et al., 2024), corpses from casework (Javan et al., 2016a; Pechal et al., 2018; Lutz et al., 2020; Zhang et al., 2021), rodent (Liu et al., 2020; Zhao et al., 2022) and pig (Pechal et al., 2014; Iancu et al., 2015, 2016, 2024)] have been tested in indoor and outdoor scenarios. Sample collection to characterise the thanatomicrobiome, and the epinecrotic microbiome generally consisted of vigorously swabbing anatomical sites, such as the oral cavity, nose, hands, the torso and rectum (Pechal et al., 2014, 2018; Iancu et al., 2015, 2016, 2023, 2024; Johnson et al., 2016; Ashe et al., 2021; Zhang et al., 2021; Zhao et al., 2022; Burcham et al., 2024), as well as collecting tissue samples from skeletal elements (ribs) (Damann et al., 2015; Deel et al., 2021) and internal organs (blood, heart, brain, liver, spleen and cecum) (Can et al., 2014; Hauther et al., 2015; Javan et al., 2016a; DeBruyn and Hauther, 2017; Liu et al., 2020; Lutz et al., 2020). Different anatomical regions of the body harbour a unique microbiome in life. In the early post-mortem period after death, these regions exhibit distinct successional shifts in microbial community diversity and abundance between body regions, tissues and organs (Can et al., 2014; Pechal et al., 2014, 2018; Javan et al., 2016a; Lutz et al., 2020). While the early post-mortem preserves the individuality of anatomical-site microbial signature, as decomposition progresses microbial communities from the gut, oral cavity and rectum migrate and colonise the body (Javan et al., 2016a; Moitas et al., 2023).

Identification of core bacterial taxa within numerous empirically validated studies lends additional support towards forming a universal network of decomposers with a finer taxonomic resolution that would ensure the estimation of more consistent time-since-intervals and the development of a reliable “microbial clock” (Metcalf et al., 2013; Lauber et al., 2014; Pechal et al., 2014; Burcham et al., 2024). For PMI estimation shifts at the phylum level (Actinobacteria, Bacteroidetes, Firmicutes and Proteobacteria) (Metcalf et al., 2013; Pechal et al., 2014; Iancu et al., 2015) have been observed providing a board overview of microbial changes during death (Table 1). Specific shifts during decomposition of taxa at genus, family and species level within these phyla require that a finer taxonomic resolution is developed for reliable post-mortem clocks using microbial communities. Specific families of bacterial taxa have been identified from the phyla Actinobacteria, Bacteroidetes, Firmicutes and Proteobacteria found in the gut/abdominal cavity (*Clostridiaceae, Lactobacillaceae, Bacteroidaceae, Xanthomonadaceae, Enterococcaceae*) (Metcalf et al., 2013; Hauther et al., 2015; DeBruyn and Hauther, 2017), on the skin [*Campylobacteraceae, Pseudomonadaceae, Sphingobacteriaceae, Streptococcaceae, Clostridiaceae, Lactobacillaceae and Xanthomonadaceae*] (Metcalf et al., 2013; Iancu et al., 2023), and in the oral cavity [*Prevotellaceae (Prevotella), Streptococcaceae (Streptococcus), Veillonellaceae (Veillonella), Micrococcaceae (Rothia)*, *Pseudomonadaceae (Pseudomonas)*, and *Moraxellaceae* (*Psychrobacter*)] (Hyde et al., 2013; Iancu et al., 2015; Javan et al., 2016b; Ashe et al., 2021; Wang et al., 2024). These taxa exhibit changes in diversity and richness during decomposition, which can be used as a microbial clock for PMI estimation (Metcalf et al., 2013, 2016). Shifts in the abundance and diversity of microbial communities have been correlated with internal biochemical changes of the body (Pechal et al., 2018; Deel et al., 2021). For instance, the decline of anaerobic bacteria, *Bacteroides* and *Lactobacilus,* has been found to coincided with the shift in conditions of the body cavity as oxygen is reintroduced post-rupture (Hauther et al., 2015), while *Pseudomonas* and *Clostridium* have been cited to release collagenases to break down bone (Deel et al., 2021). The “Post-mortem Clostridium Effect” (PCE), a concept introduced by Javan et al. (2017) refers to the ubiquitous nature of C*lostridium* spp. found throughout decomposition, making it a key microbial marker in the PMI. The effect is characterised by the rapid colonisation of the body by this species as conditions become more anaerobic (Can et al., 2014; Iancu et al., 2016; DeBruyn and Hauther, 2017; Liu et al., 2020) and due to their proteolytic function for breaking down collagen (Javan et al., 2017). A diverse range of C*lostridium* spp. have been characterised in the early post-mortem period from the thanatomicrobiome at 4 h (112), 12 h (Iancu et al., 2023), as well as at 24h and 58 h (Can et al., 2014) (Table 2), making it an essential biomarker for PMI estimation.

Complex algorithms leveraging machine learning (Table 2) (Johnson et al., 2016; Metcalf, 2019; Liu et al., 2020; Cui et al., 2022; Yang et al., 2023), allows for interpretation of the microbiome composition data through diversity and richness calculations, as well as the development of predictive modelling through machine learning algorithms (random forest regression, xgboost method and neural networks) (Pechal et al., 2014, 2018; Johnson et al., 2016; Liu et al., 2020; Zhao et al., 2022; Burcham et al., 2024; Iancu et al., 2024). These models have demonstrated robust performance, with accuracy assessed by metrics such as mean absolute error (MAE), which quantifies the average deviation between predicted and actual PMI values. Studies have shown that random forest models built on microbiome data from skin, organ and in some cases gravesoil samples, particularly using 16S rRNA gene markers, provide reliable PMI estimates, often within a small error margin over decomposition periods of up to several weeks. This has been demonstrated using mouse models where the PMI was estimated within approximately 3 days over a period of 48 days (Metcalf et al., 2013, 2016). Models leveraging random forest regression algorithms appear to be the preferred machine learning method used for PMI estimation (DeBruyn and Hauther, 2017; Liu et al., 2020; Lutz et al., 2020; Deel et al., 2021; Zhang et al., 2021; Zhao et al., 2022; Burcham et al., 2024; Iancu et al., 2024). Random forest algorithms are based on supervised learning and use multiple decision trees to make predictions (Berk, 2008). Random forest have effectively been used for PMI estimation because they can work with and process large datasets (Pechal et al., 2018; Zhang et al., 2021; Burcham et al., 2024), reduce errors, increase reliability for PMI estimation (Pechal et al., 2018; Namkung, 2020; Li et al., 2023; Wu et al., 2024) and allow for the integration of various multi-omics datasets (Burcham et al., 2024; Li et al., 2024). In principle, each decision tree is constructed from different subsets of microbiome sequencing data, capturing patterns in the microbial taxa present in the samples (Namkung, 2020; Schonlau and Zou, 2020). The algorithm combines the outputs into a final prediction. Despite growing interest, the predictive performance of random forest models remains variable across studies, species, and sampling strategies. Using a mouse model Liu et al. (2020) predicted PMI from microbial communities in the internal organs with a high accuracy of 1.5 ± 0.8 h within 24-h decomposition and 14.5 ± 4.4 h within 15-day decomposition. Yet in human cadavers Deel et al. (2021) reported a MAE of 724 to 853 ADD ± 39 days over a total of 5,201 ADD, and a MAE of 793.33 ± 34 days over two seasons using multiple ribs. Yang et al. (2023) further highlighted seasonal affects from swab samples collected from the rectum and gravesoil of pig carcasses. The winter trail rectal samples yielded a MAE of 2.478 days, while gravesoil performed slightly better with a MAE of 2.001 days. In summer, rectal samples had a MAE of 1.375 days and the gravesoil sample had a MAE of 1.567 days (Yang et al., 2023). In a severe cold environment Iancu et al. (2024) found the best model for PMI prediction, combined internal and external swabs of pig carcasses for a MAE of 1.36 weeks. It is worth noting that the cross-validation of predictive models improve the accuracy and objectivity of PMI estimates by minimising human bias, while training and test datasets add to the robustness of the findings (Johnson et al., 2016; Pechal et al., 2018; Liu et al., 2020; Deel et al., 2021; Hu et al., 2021; Zhang et al., 2021; Burcham et al., 2024; Iancu et al., 2024). Leveraging similar machine learning approaches for gravesoil microbiome signatures could be applied to the estimation of the PBI and PTI.

### 3.2 Microbial succession for post-burial and post-translocation interval estimation

Anthropogenic activities, such as the act of burying and translocating a body, can disturb the natural stratigraphy of soil, impacting ecosystems and microbial communities (Jansson et al., 2023) to accommodate the needs of humans. Studies of Vindolanda, a Roman auxiliary fort in the UK, revealed the significance of the interplay between human intervention in the environment, local ecological conditions and soil microbial communities, and the lasting impact it can have (Driel-Murray, 2001; Birley, 2009; Orr et al., 2021). For example, microbial analysis by Orr et al. (2021) revealed that soils dating to the earliest occupation of the Vindolanda site were dominated by the phyla Bacteroidetes, Firmicutes and Proteobacteria, and contained better preserved artefacts when compared to control soils, which were characterised by increased abundances of Acidobacteria, Actinobacteria and Planctomycetes. This study highlights the interconnectedness of microbial community shifts and human activity in combination with the unique environmental conditions, which collectively led to better preservation at Vindolanda. Similarly, a study from Western Kazakhstan showed the long-term impact of human intervention on soil microbial communities, specifically relating to palaeosoils below a burial mound, dating to 2,500 years ago (Kichko et al., 2023). In contrast to surface control soils, the specific burial conditions, including reduction of air, water and organic material in buried soils, led to decreases in the abundances of Actinobacteria clades of *Gaiella, Solirubrobacteriales,* and *Frankiales*. Conversely, there were increases in the diversities of Actinobacteria (*Acidimicrobiia, Propionibacteriales, Micromonosporales, Euzebyales*), Firmicutes (*Bacilli*), Chloroflexi (*Thermomicrobiales*), Acidobacteria (*Subgroup 6*), and Proteobacteria (*Tistrellales*) (Kichko et al., 2023). These studies are examples of how microbial communities in soils impacted by human intervention have distinctive compositions that differ from the microbial communities found within natural soils in that specific environment, with no human impact. Moreover, these studies highlight that the occurrence, distribution and abundance of these distinctive microbial communities are influenced by specific environmental and burial conditions.

The decomposition of a body has a similar effect on soil microbial communities. Shifts in soil microbial composition for surface depositions of mammalian or human donor cadaver remains have been reported in studies conducted in China (Guo et al., 2016; Yang et al., 2023), and the USA (Lauber et al., 2014; Cobaugh et al., 2015; Weiss et al., 2016; Burcham et al., 2024). Studies considering soil microbial shifts for buried human, pig or rodent (mice or rats) carcasses have been conducted in China (Zhang et al., 2021; Cui et al., 2022; Yang et al., 2023; Wang et al., 2024), the USA (Keenan et al., 2018), and the UK (Olakanye et al., 2017; Olakanye and Ralebitso-Senior, 2018; Olakanye and Ralebitso-Senior, 2022; Procopio et al., 2019; Bisker et al., 2021, 2024). Underpinning these studies is the focus to develop more reliable methods for PMI estimation by using gravesoil, sometimes complemented by the analysis of the post-mortem human microbiome (Lauber et al., 2014; Yang et al., 2023; Burcham et al., 2024). Similar to predicting PMI from the thanatomicrobiome and epinectrotic microbiome, PMI estimation from gravesoil is also sensitive to species (rodent, pig and human) and seasonal context. Zhang et al. (2021) used gravesoil microbial community from buried rat models to achieve a MAE of 2.04 ± 0.35 days, which improved to a MAE of 1.82 ± 0.33 days when the biomarker set was considered during 60-day decomposition. Cui et al. (2022) refined this approach by focusing on the 18 dominant genera from buried mice to obtain an MAE of 1.27 ± 0.18 day within 36 days. Seasonality affected the generalizability of the models. Yang et al. (2023) found that gravesoil from buried pigs produced a MAE of 1.567 days for summer, but the accuracy decreased for winter with a MAE of 2.001 days. Wang et al. (2024) investigated a different effect by introducing fresh and buried pig femurs and reported a MAE of 55.65 ADD. Placed in the correct interpretive frame, the results of studies analysing gravesoil yield meaningful information about the dynamics of microbial shifts in clandestine graves, and the PBI. The analysis of gravesoil in these studies, alters the parameter of interest (specifically the biological process being captured and the timeframe being estimated). Rather than providing an estimate of the PMI, as is commonly assumed, this experimental design directs researchers toward estimating PBI instead. Previous studies have undoubtedly laid an important foundation for PMI estimation, and their methodological contributions and statistical analysis remain valid. However, the concern arises from how their findings have been interpreted. Since soil microbial communities shift after the inclusion (deposition or burial) of mammalian remains (and not at the start of death unless death occurs at the exact same time and place), the presented evidence about PMI is, in fact, evidence for PBI estimation. An example of the difference between the PMI and PBI has been highlighted by Damann et al. (2015) who reported the two intervals, the first is the interval between the time of death and sample collection (the PMI), and second is the interval between placement and sampling (the PBI). As illustrated in the study, the PBI is shorter than the PMI as it begins once a body is deposited (or buried), with microbial change in the burial environment shifting at the moment of placement and not at death.

Estimating the PBI and PTI relies on characterising non-native microbial taxa in soil, which serve as markers to distinguish natural soils from gravesoils (Tables 3, 4). During decomposition, microbial communities will migrate into the soil, exploiting the resources that are available and forming a microbial community that is unique to that CDI (Weiss et al., 2016). Changes in bacterial community structure over time and season for buried pig tissue and plant litter samples have been observed by Olakanye and Ralebitso-Senior (2018). Changes were recorded for the phyla Actinobacteria (*Micromonosporaceae*), Bacteroidetes (*Sphingobacteriaceae*), Firmicutes (*Planococcaceae*) and Proteobacteria (*Rhizobiaceae*, *Hyphomicrobiaceae* and *Xanthomonadaceae*) with unique microbial shifts persisting up to day 365 after burial. Control soils were characterised by Actinobacteria *(Nocardioidaceae)*, *Firmicutes* (*Alicyclobacillaceae*), and Proteobacteria (*Comamonadaceae* and *Bradyrhizobiaceae*). Microcosms containing pig tissue were characterised by Actinobacteria (*Nocardiaceae and Micrococcaceae*), Proteobacteria (*Alcaligenaceae and Hyphomicrobiaceae*) (Olakanye and Ralebitso-Senior, 2018). These findings are also consistent with Procopio et al. (2019), who identified that mammal-derived Bacteroides (*Bacteroidacea*) could be identified in grave soils collected directly next to the superior part of the carcass, and distinguished from control soils 6 months post-burial. Human-derived Bacteroides have also been detected in soils collected from underneath the body, 198 days after cadaver surface placement (Cobaugh et al., 2015). Other studies have reported the existence of decomposition-related microbial taxa at post-burial intervals of 120 days (Wang et al., 2024) for soils collected from pig femurs burials and 720 days from homogenised soil samples collected at 4 sides of the grave (Bisker et al., 2021). These findings indicated further that non-native taxa do persist in gravesoils and that they might serve as a universal microbial marker for buried remains, demonstrating the value of using microbial succession. Studies have attempted to map shifts in microbial composition over several years to determine whether gravesoils return to basal levels after decomposition. Singh et al. (2018) showed that decomposition-impacted soils from 0 to 10 cm below human cadavers did not recover to basal levels even after 732 days, reflecting similar findings by Cobaugh et al. (2015). A second study by Keenan et al. (2018) reported on the impact of human cadaver decomposition on the soil microbial communities and soil composition, which still measurable after 4 years. Additionally, in the same study human-associated Bacteroides was still detectable at the bottom of the grave (Keenan et al., 2018), reflecting similar findings by Cobaugh et al. (2015). The Burcham et al. (2021) study highlighted that faint microbial signatures from soils collected directly underneath cadaveric remains could be used to differentiate gravesoils from natural soils after 10 years. The strongest distinction between gravesoils and natural soils was up until 12 months after deposition, after which the soil microbial communities began to return to basal levels (Burcham et al., 2021).

The decomposition of mammalian remains has a lasting spatiotemporal effect on soil microbial communities (DeBruyn et al., 2024; Taylor et al., 2024), which can potentially be used as markers for PBI estimations. The persistence of specific bacterial phyla, such as Acidobacteria, Firmicutes, and Proteobacteria for extended periods post-burial in gravesoil, underscores their potential as indicators for mammalian decomposition and for PBI estimation. Given these studies leveraging gravesoil two things are clear: first the decomposition of mammalian remains has a lasting spatiotemporal effect on soil microbial communities (DeBruyn et al., 2024; Taylor et al., 2024), which can be used as markers for PBI estimations; and secondly currently for the extended burial period, gravesoil identification relies primarily on the detection, inclusion, or persistence of specific microbial taxa that differ from the background, undisturbed soil community. For more reliable PBI estimations, research should prioritise the development of models that incorporate finer taxonomic classifications, beyond phylum and genus. Additionally, further research and results need to be tested and evaluated across different biogeographic locations and burial conditions. For more reliable PBI estimations, research should prioritise the development of predictive regression models.

Although few cases involving the translocation of single clandestine graves are published, the PTI is an important time-since-interval and can provide valuable information to forensic investigators regarding the context of the crime, body disposal patterns and treatment of a victim after death. The need to investigate the translocation of remains, and hence for PTI estimations, has been highlighted in previous publications. In their paper discussing the use of ninhydrin reactive nitrogen in soil to detect graves, Carter et al. (2008) stated that *“Bodies can be moved from the original site of death (and subsequent scenes).”* As such, depending on when the body was moved and translocated from the original burial or deposition site, it is possible that the *“removed human may leave a persistent effect in former gravesoil*” (Carter et al., 2008). The persistence and uniqueness of microbial communities from the human microbiome that are found in soil have also been posited by Cobaugh et al. (2015) as a forensic tool which could “*prove useful in cases where body remains have been moved from the original location of decomposition.”* Considering this, the potential of shifts and the persistence of soil microbial communities during the decomposition process and after translocation, can offer further insight as a “microbial clock” beyond PMI estimations (Metcalf et al., 2013) to estimate the PBI and PTI. Ralebitso-Senior et al. (2016) proposed that the microbial communities found within gravesoils could be used as a means to link a victim to a crime scene, which could be especially useful in instances where “*remains have been moved and/or decomposed*.” Building on this concept, it is also possible that the PTI could provide evidence linking suspects to both primary and secondary locales as crime scenes, and at specific temporal intervals such as time of deposition or burial. Fu et al. (2019) also stated that the *“dissimilarity in soil communities may help experts to identify the original location from which a cadaver has been moved*.” Although Gemmellaro et al. (2023) referred specifically to fungal communities, their recommendation highlights the need for further research to investigate how the translocation of buried mammalian carcasses and human donor cadavers affects the decomposition process and the microorganisms that drive it. Therefore, there is potential for soil microbial communities to not only provide a post-mortem time-since-interval for when remains were translocated but also to aid investigators in narrowing down the original location the remains were moved from if discovered at the secondary locale. The use of multidisciplinary approaches such as forensic ecogenomics, forensic archaeology, and forensic geology to determine when remains were intentionally recovered and reburied by perpetrators, would offer insight into creating a potential timeline of events, which can aid investigators in linking suspects to specific sites and crime scenes, aiding the investigation and prosecution process (Dirkmaat and Cabo, 2016; Ralebitso-Senior et al., 2016).

The utility of microbial communities in forensic investigations also lies in their ability to provide valuable information about post-mortem treatment of the body and a timeline of events after death, and becomes useful in cases where bodies have been moved (Demanèche et al., 2017; Karadayı, 2021). In their study using surface deposition of human donor cadavers, Cobaugh et al. (2015) reported increases in the abundance of Proteobacteria and Firmicutes, while Acidobacteria abundance decreased during active decay. The researchers argued that microbial communities from the human microbiome, including Actinobacteria (*Eggerthella*), Firmicutes (*Phascolarctobacterium and Tissierella*) and Proteobacteria (*Paenalcaligenes*), that were introduced into the surrounding soil during decomposition, would not persist for long outside of their natural environment. This study found that once the dry remains were removed from the site, there was a decrease in microbial community abundance. It was also found that members of the genus *Bacteroides* (human-associated) persisted within the CDI 198 days after cadaver deployment on site. Once the dry remains were removed from the deposition site, there was a decrease in their abundance by day 126 and taxa was not detectable in the grave by day 204 (Cobaugh et al., 2015). Subsequently, a 180-day study by Olakanye and Ralebitso-Senior (2022) characterised microbial shifts in gravesoil mesocosms before and after the exhumation of whole piglets and demonstrated that microbial community structure and composition can be used in PTI estimation. Their study showed changes at 150 days post-burial at which point Proteobacteria *(Xanthomonadales and Xanthomonadaceae)* and Verrucomicrobiota *(Verrucomicrobiaceae)* were abundant. On the other hand, Bacteroidetes (*Bacteroidales*), Firmicutes (*Clostridiales and Clostridiaceae_1*) and Proteobacteria (*Hydrogenophilales and Hydrogenophilaceae*) were abundant in homogenised soils samples collected from random mesocosm positions 120 days after exhumation, indicating a potential decomposer network for translocated remains and PTI estimation (Olakanye and Ralebitso-Senior, 2022). Considering the impact of a decomposition event, which alters the biochemical signature of soils (Benninger et al., 2008; Macdonald et al., 2014; DeBruyn et al., 2021), the burial and exhumation of mammalian remains will induce specific shifts in the composition of the soil microbial community throughout the post-mortem period, driven by the decomposition process (Olakanye et al., 2014, 2015). This allows a unique gravesoil microbial community to develop which will be distinct from microbial communities in the natural background soil. Similar to how environmental conditions aided in the uniqueness of microbial communities in Vindolanda and Western Kazakhstan, surface depositions and subsurface burials can preserve microbial signatures, allowing them to be used as evidence in forensic investigations, for the estimation of PBI and PTI, and as markers to distinguish natural control and gravesoils.

## 4 Framework for PBI and PTI

**Figure 5:**
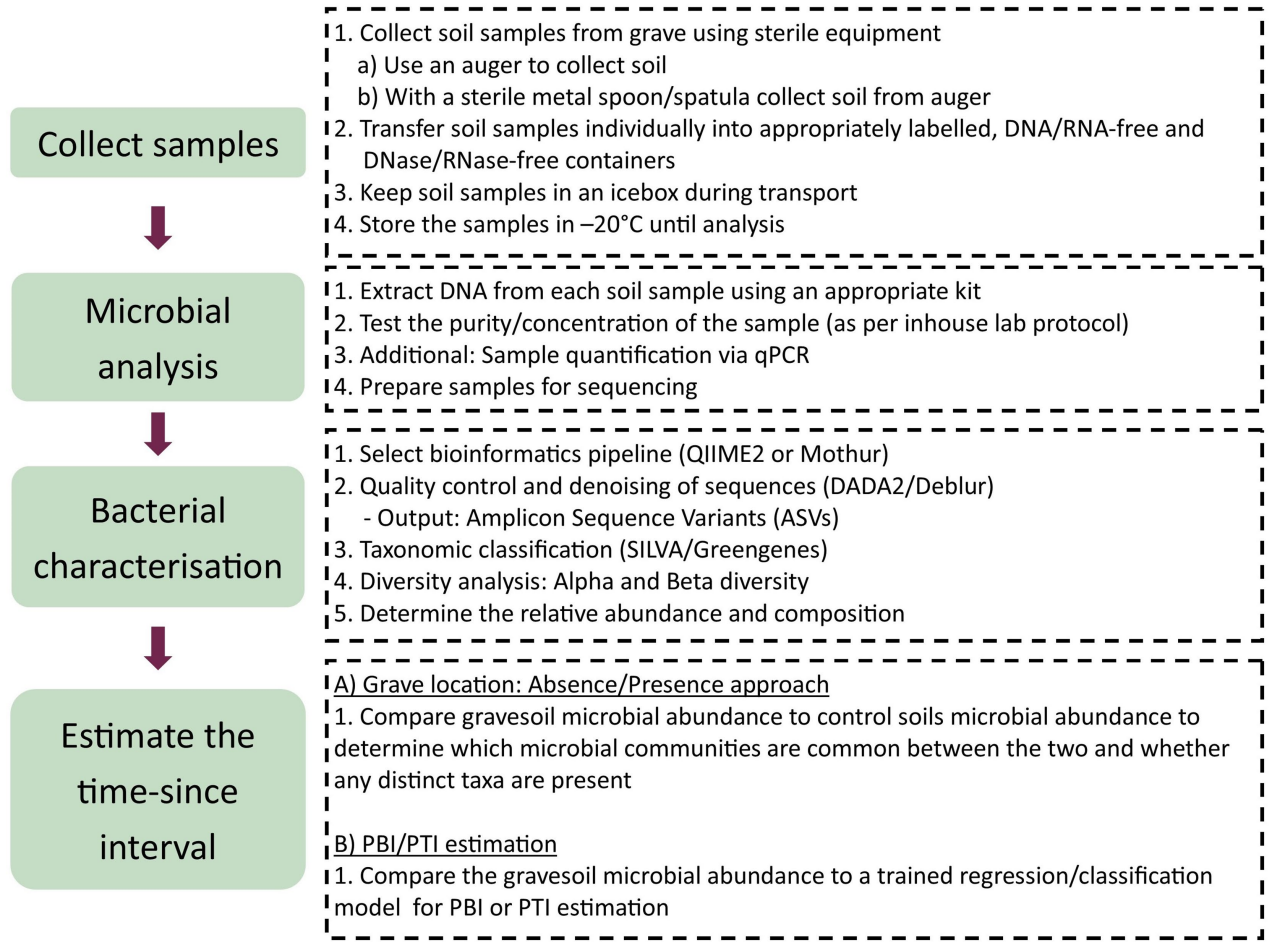
Conceptual framework for using gravesoil microbial communities to estimate the PBI and the PTI. This framework is modelled after the Pechal et al. (2014) framework to estimate PMI using microbial communities from the body.

The dynamics of, and shift in, gravesoil microbial communities can provide a substantial contribution to the estimation of the PBI and PTI. Specifically, the distinct microbial communities resulting from decomposition provide a reliable means of identifying grave sites. The temporal persistence of these microbial communities in terrestrial burial environments indicates that they could be a potential tool in the estimation of post-mortem time-since-intervals for forensic investigations and aid in clandestine grave location. Just as these communities can be used to estimate the PMI, they could also serve as a post-mortem “microbial clock” to estimate the time-since-burial as well as the time since a body was removed from its burial or depositional environment. This approach leverages the same principles used for time-since-death estimation using the microbiome, extending their application to scenarios involving intentional exhumation and translocation of remains in forensic cases. We propose a framework (Figure 5) showcasing how microbial communities from gravesoil can be incorporated into case work to estimate the PBI and PTI.

Once a clandestine grave site is located, investigators would collect soil from the grave pits. For graves that contain a body or skeletonised remains, *in situ* soil samples can be collected from around and underneath the remains using sterilized soil corers or metal spatulas. In the case of empty grave pits soil samples can be collected from the bottom of the grave. To avoid contamination of the crime scene and grave environment, any tools used in the excavation and recovery of the remains must be sterilised before and after use according to the preferred protocol of the research laboratory or crime investigation unit. Soil should be collected from 4 cardinal points in and at the centre of the grave, to ensure a representative sample of the entire microbial community at the time the grave is discovered. Sterile sample tubes that are DNA/RNA-free and DNase/RNase-free need to be labelled clearly with the case number, location and sample date. Triplicate control soil samples can be collected from around the grave site at 2 m, 5 m, and 10 m intervals to serve as reference samples for the site from undisturbed natural areas. On-site and during transport, all the samples must be packed individually and kept in an icebox. Once in the laboratory, the samples can be stored in a − 20 °C freezer until analysis. Control soils need to be sieved through 2 mm mesh to remove any twigs, small stones, or debris, while ensuring no contamination from the laboratory environment. After microbial DNA extraction, the samples can be prepared for 16S rRNA sequencing through MPS. During the bioinformatic pipeline, sequences should be classified, and the microbial composition and relative abundance determined.

At this stage, the sequence data can be used to determine based on the presence or absence of microbial biomarkers whether a sample comes from a human-derived gravesoil , with reference to previously published data for the specific environment and conditions (Tables 3, 4). The sequence data and microbial relative abundances in the samples can also be used to estimate the PBI and PTI. This can be done by comparing the community abundance of the sample to a trained regression/classification model, such as a machine learning random forest model. While the regression and classification models central to this approach are still under development, the framework is grounded in established principles of machine learning, forensic ecology and forensic ecogenomics from previous studies (Johnson et al., 2016; Liu et al., 2020; Burcham et al., 2024). As a conceptual tool, it highlights a path for future empirical research. By outlining the process from sampling to predictive modelling, the proposed framework aims to bridge the gap in current post-mortem time-since-interval estimation, specifically for the use of gravesoil to contribute to the PBI and the PTI.

**Table 3:** Study matrix summarizing experimental models, environmental conditions, sequencing approaches, and key findings in reviewed studies from gravesoil microbial communities.

Study	Characteristics of the burial environment	Sampling location and days	Key taxa shifts	Findings and accuracy
Cobaugh et al. (2015) Human donor cadavers (*n* = 4) PBI: 83–198 days	Burial conditions: Placed on the surface Environment: University of Tennessee Anthropology Research Facility, Knoxville, Tennessee, USA. Temperate deciduous forest, well-drained fine-textured clayey soil Summer and fall seasons	Sample: Ceacum swabbed before placement through a small incision, soil samples collected prior to placement Control: Control soils collect alongside experimental soils Sampling time: 8 sampling periods: Initial, bloat, bloat-active, Active, Advanced active, Advanced I, Advanced II and Advanced III	Acidobacteria, Nitrospira, Verrucomicrobia and Armatimonadetes abundant before cadaver placement but declined during later decomposition. Planctomycetes decreased during Bloat-Active to Advanced decay II, but returned during Advance III. Firmicutes increased during decomposition but decreased during Advanced III Human microbiome: Active decay: Increase in *Bacteroides, Staphylococcus and Enterococcus.* Advanced decay: Increase in *Lactobacillus, Phascolarctobacterium, and Eggerthella* Bacteroides persisted within soil for 198 days post burial. Deline in Bacteroides after dry remains were removed (PTI). Decrease in abundance by day 126 and taxa undetectable by day 204	The soil microbial community is impacted by cadaver placement Human-derived Bacteroides survives in soil outside of the body and persists for sometime after remains are removed from the site
Weiss et al. (2016) Pigs (*n* = 4) PBI: 9 days (144 ADD)	Burial conditions: Placed 5 m apart on the surface on a polypropylenemesh frame Environment: Mead, Nebraska, USA. Grassland. Soil: Silty, clay loam (15.1% sand, 53.6% silt, and 31.3% clay) Summer season	Sample: Gravesoil collected underneath carcasses (0–5 cm) using a soil probe Control: Control soils. Sampling time: 0, 1, 2, 4, 5 6, 9, and 15 days post-mortem (day 3 and 8 skipped due to thunderstorms)	*Candidatus Chthoniobacteraceae* dominated all soils during early decomposition but decrease as remains decayed. Taxa *Gaiellaceae, Acidobacteria, and Rhodoplanes* also decreased during decomposition. Increase in taxa *Planococcaceae*, *Sporosarcina* sp., *Ignatzschineria* sp., and *Chitinophagaceae* as decomposition progressed	The presence of bacterial communities can distinguish between the gravesoil and the control soils There is a difference in the microbial communities depending on the size of the associated carcass (1 kg vs. 50 kg)
Olakanye et al. (2017) Stillborn piglets, leaf litter and control gravesPigs (*n*=3), oak leaf litter (*n*=3), and control burials (*n*=3) PBI: 270 days	Burial conditions: Each pit was 50 cm × 30 cm × 40 cm, and 2 m apart Environment: Site near North Yorkshire, UK. Soil: Loam soil constituted by (w/w) 22% clay, 32% silt and 46% sand. Winter, spring, summer, autumn	Sample: 4 soil samples collected from each pit, that was homogenised Control: Control soils from control burials Sampling time: Collected monthly	Dominant phyla: Proteobacteria, Acidobacteria, Verrucomicrobia, Bacteroidetes and Actinobacteria Day 180: Decrease in abundance of Acidobacteria_Gp6_order (7.02–14.44%) between the control and treatments. Planctomycetales dominant in pig burials (treatment). Anaerolineales and Acidobacteria_Gp7_- order increased in leaf litter soil D210: Acidobacteria_Gp6_order and Acidobacteria_Gp16_order increased for all samples. Planctomycetales abundance decreased D240: Increase in abundance of Methylococcales and Anaerolineales in leaf litter soil D270: Increase in abundance of Xanthomonadales and a decrease of Acidobacteria_Gp6_order for control soils	Gravesoils from pig burials could be distinguished from leaf litter soil
Keenan et al. (2018) Human donor cadaver (*n* = 3) PBI: 4 years	Burial conditions: Two grave pits (~2 m × 2 m × 0.7 m); mass grave with 3 individuals and empty control grave Environment: University of Tennessee Anthropological Research Facility, Tennessee, USA. Temperate mixed deciduous forest. Soil type: Plant material and loam in the O-A horizons (0–10 cm), underlain by clay loam and channery clay loam extending to bedrock (limestone, shale, and sandstone)	Sample: Destructive sampling through excavation; samples collected from around the grave (linearly moving away from pit), inside the grave and under the remains Control: soils from control burial Sampling time: Single collection at destructive sampling	Human-associated *Bacteroides* were not detected in the transects, but were detected in the bottom grave after 4 years	Human-associated *Bacteroides* persist in graves under remains for 4 years
Olakanye and Ralebitso-Senior (2018) Pig tissue (*n* = 24), leaf plant litter and control soil (80 g) PBI: 365 days	Burial conditions: Outdoor microcosms Environment: Sieved soil collected from Bishop Burton College of Agriculture, Lincolnshire, UK July 2013 (summer), January 2014 (winter) and July 2014 (summer)	Sample: Destructive sampling Control: Control soils Sampling time: 7, 14, 28, 60, 120, 180, 300 and 365	Actinobacteria, Proteobacteria, Bacteroidetes and Firmicutes are dominant in all microcosms. *Sphingobacterium* and *Pedobacter* are dominant in the pig soil. While *Rhodanobacter* and *Shinella* are dominant in the plant litter soil Day 0: Proteobacteria and Alphaproteobacteria (*Hyphomicrobium*) are dominant Day 28: Rhizobiaceae increased in plant litter soil. Planococcaceae and Micromonosporaceae increased in soil containing pig tissue Bacteroidetes increased by Day 365 for the soil containing pig tissue	Several taxa identified that can be used as biomarkers to distinguish soils from pig and plant litter seasonally
Procopio et al. (2019) Pig (*n* = 4) PBI: 1–6 months	Burial conditions: Buried in 40 cm deep gravepits Environment: HuddersFIELD outdoor taphonomy facility, University of Huddersfield, UK May–November 2016 End of spring to the end of autumn	Sample: Destructive sampling; samples collected from around the remains and bagged together Control: Control soils taken at the same depth but in areas with no pig remains Sampling time: 1,2,4, and 6 months	Proteobacteria were the most abundant, followed by Bacteroidetes, Acidobacteria, Actinobacteria, and lastly by Firmicutes. Pig burials: Proteobacteria (Xanthomonadaceae and Alcaligenaceae) increased in their abundance, but at 4 months post-mortem, Bacteroidetes (Flavobacteriaceae) increased in abundance as decomposition progressed. Sphingobacteriaceae were abundant after the first month but decreased after 6 months. Firmicutes increased in the later deposition stages. Control burials: Dominant by Acidobacteria followed by Proteobacteria.	Shifts in gravesoil microbial communities can be distinguished from natural control soils
Bisker et al. (2021) Pig (*n* = 3), oak leaves (*n* = 3), control burials (*n* = 3) PBI: 24 months	Burial conditions: Triplicate burials with either piglet or leaf litter. Pigs placed in wire mesh before burial Environment: North Yorkshire, UK. Woodland (oak trees). Soil: Clay (22%), silt (32%) and sand (46%) December 2014–December 2015	Sample: Soil samples collected from 4 regions of each grave (at 20–60 cm depth) Control: Samples from control burials Sampling time: Sampled monthly for 12 months, and again at 24 months	Acidobacteria, Proteobacteria, Firmicutes, Planctomycetes, and Chloroflexi were dominant in year 2 of the pig burial soils Methylococcales, Sinobacteraceae, Candidatus, and Flavobacterium, found in plant and pig burials, compared to control soils Family: Most abundant taxa for extended PBI: RB40_family, mn2424_family, Chloroflexi, Chtoniobacteraceae, Hyphomacrobiaceae, Pirellulaceae, Chitinophagaceae, Gemmataceae, Sinobacteraceae, Gaiellaceae, and Cytophagaceae.	Phylum and genus level classification can distinguish pig and plant litter burials from control soils, but a finer resolution is needed to distinguish between plant litter and pig burials
Burcham et al. (2021) Pigs (*n* = 2) PBI: 10 years	Burial conditions: Placed on the soil surface, wire cages placed above pigs Environment: Benton County near Philomath, Oregon, USA. Temperate coniferous forest, consisting of Vine Maple, Ocean Spray, salal, and bracken fern, and with soils are characterized as the Price-MacDunn- Ritner soil series complex	Sample: Soils collected with a centrifuge tube underneath and next to carcasses at 4 location: 2 control samples from sites 10 m away (north and south), one sample 1 m north of the carcass, and one sample underneath the carcass Control: Soil samples collected on site Sampling time: Over 10 years: 2 weeks (pig 1 only), 1 month, 6 months, 1 year, 2 years (pig 2 only), 6 years, 7 years, 10 years	*EB1017* genus and *Chthoniobacter* decreased underneath the carcass from 0–24 months. From 24 to 84 months post-mortem *Chthoniobacter* increased in abundance underneath the carcass. From 1–12 months post-mortem *Rhodospirillaceae* genus decreased, but increased in abundance from 24–84 months. *Devosio* increased in abundance from 6–24 months post-mortem, but decreased underneath the pig carcass from 24–84 months	Distinct gravesoil microbial communities can be distinguished from control soils 10 years after pig cadaver placement
Zhang et al. (2021) Rats (*n* = 50) PBI: 60 days	Burial conditions: Buried 20 cm deep in an open space Environment: Shanxi Medical University, China	Sample: Destructive sampling at each time point. Sterile swabs are used to collect samples from the gravesoil, rectum and skin Control: Five sterile swabs Sampling time: Day 0.5, 1, 2, 3, 7, 14, 30, 45 and 60	Day 0: Proteobacteria, Acidobacteria, Chloroflexi and Actinobacteria were dominant Day 60: Bacteroidetes, Firmicutes and Proteobacteria were the dominant phylum. At the family level, the family Bacillaceae, Flavobacteriaceae, Alcaligenaceae, Sphingobacteriaceae and Caulobacteraceae were dominant.in later post-mortem period	At the beginning of decomposition, the 3 sites had distinct microbial community abundance. During later decomposition, the abundance becomes similar across all sites Gravesoil provides the most accurate prediction All OTUs: Gravesoil: MAE of 2.04 ± 0.35 days Rectum: MAE of 2.24 ± 0.38 days Skin: MAE of 2.15 ± 0.40 days Models with biomarker set: Gravesoil: MAE of 1.82 ± 0.33 days Rectum: MAE of 2.06 ± 0.38 days Skin: MAE of 2.12 ± 0.40 days
Cui et al. (2022) Mice (*n* = 65) PBI: 36 days	Burial conditions: Buried individual in single graves (20 cm × 20 cm × 20 cm) Environment: Forest with loose soil	Sample: 5 mice were destructively sampled every 3 days. Gravesoil collected from under buried carcasses Control: Day 0 soils Sampling time: Days 0, 3, 6, 9, 12, 15, 18, 21, 24, 27, 30, 33, and 36	Dominant taxa: Proteobacteria, Acidobacteria, Actinobacteria, Chloroflexi, Nitrospirae, Bacteroidetes, Thaumarchaeota, Gemmatimonadetes, errucomicrobia, Firmicutesand Latescibacteria Proteobacteria and Bacteroidetes increased during decomposition, while Acidobacteria, Actinobacteria, Chloroflexi and Nitrospirae decreased Pseudomonas is dominant genera. Oxalobacteraceae, members of the family Comamonadaceae, (*Vitreoscilla and Sphingobacterium*) abundance increased during decomposition, while Gemmatimonadaceae, RB41 (subgroup 4), Roseiflexus, GR-WP33-30, Xanthobacteraceae and MB-A2-108 decreased	Soil samples from different PMI can be separated from each other MAE of 1.27 ± 0.18 days within 36 days
Olakanye and Ralebitso-Senior (2022) Pig (*n* = 1), control (*n* = 1) PBI: 180 days (6 months) PTI: 120 days (4 months)	Burial conditions: Mesocosms consisting of stillborn pig and soil control burial (empty) Environment: Homogenised soil (sandy clay loam) from Framwellgate Moor, County Durham, UK November 2014 (late autumn–winter) to September 2015 (early autumn)	Sample: Random soil samples collected Control: Soils from the control burial Sampling time: At 2 and 4 weeks, then monthly for a total of 10 months	Pre-exhumation and post-exhumation dominant Phylum: Proteobacteria, Acidobacteria, Bacteroidetes, Verrucomicrobia, Actinobacteria and Planctomycetes Pre-exhumation – Day 14: Increased abundances of Pseudomonadales and Flavobacteriales Day 60 dominated by Pseudomonadales, Flavobacteriales, Burkholderiales, and Campylobacterales in the piglet mesocosm Soil only control mesocosm dominated by were predominance of Acidobacteria_Gp6_order, Spartobacteria order, Planctomycetales and Rhizobiales Day 150 with increased Xanthomonadales, Burkholderiales, Nitrosomonadales, Sphingobacteriales, and Flavobacteriales in the piglet mesocosm On Day 210 (30 days after grave exhumation) decreased dominances in piglet mesocosm of Xanthomonadales and Burkholderiales, while Sphingobacteriales, Verrucomicrobiales and Sphingomonadales increased in abundance Xanthomonadales subsequently showed its highest abundance day 240 (60 days after the pig exhumation) Day 270 increased abundances of Flavobacteriales and Alphaproteobacteria_order but decreased abundance of Xanthomonadales of the piglet mesocosm Day 300 (120 days since exhumation) increased abundances of Hydrogenophilales, Clostridiales, Bacteroidales, and Flavobacteriales	After exhumation (day 180) experimental and control soil could be distinguished from each other based on family level characterization Seasonal changes had an effect on the microbial activity over time
Yang et al. (2023) Pig (*n* = 3) PBI: 32–40 days	Burial conditions: Placed in a shallow grave Environment: Animal Care and Use Committee of the Nanjing Agricultural University, Nanjing, China Winter and summer seasons	Sample: Rectal samples were collected with swab, gravesoil samples were collected with a sampler Control: Day 0 samples Sampling time: Winter sampling (40 days): 0, 8, 16, 24, 32, and 40. Summer sampling (32 days): 0, 8, 16, 22, and 32	Phylum: Firmicutes and Bacteroidota were the abundant in both the winter and summer from rectum samples. Proteobacteria was more abundant in the rectum samples collected in winter for late decomposition Winter: The genera *Vagococcus, Myroides,* and *Carnobacterium* Summer: The genera *Proteus, Candidatus_Soleaferrea, Tepidimicrobium, Savagea,* and *Sporosarcina*	Seasonality has an impact on the microbial community succession, which can impact PMI estimation Winter pig rectal with MAE of 2.478 days Winter pig soil samples with MAE of 2.001 days Summer pig rectal samples with MAE of 1.375 days Summer pig soil samples with MAE of 1.567 days
Bisker et al. (2024) Mice (*n* = 18) PBI: 360 days	Burial conditions: Outdoor burial site consisting of U-PVC pipe microcosms. 3 × triplicate mice cadavers, decomposing on the surface, in the subsurface (10 cm) and soil-only Environment: Teesside University crime scene house, Middlesbrough, UK	Sample: Samples taken with a metal spatula at depths: 0–10 cm, 30, 60, and 90 cm Control: Soil-only controls Sampling time: Day 1 and 15, and then at 30-day intervals up to 360 days	Dominant taxa at Phylum level for all samples: Proteobacteria, Planctomycetota, Bacteroidota, Actinobacteriota, Acidobacteriota, Chloroflexi, Verrucomicrobiota, Myxococcota, Halobacterota and Bdellovibrionota Dominant taxa at genus level for all soil samples: Pir4 lineae, Rhodanobacter, Allocateliglobosispora, Chryseolinea, Chthoniobacter, SH-PL 14, Pseudolabrys, Devosia, Pirellula, and Methanosarcina Variovorax was more abundant in the control soils	Microbial communities could distinguish between control and experimental soils. However, finer resolution is needed to distinguish surface from subsurface soils
Wang et al. (2024) Unfrozen pig femurs (*n* = 10) PBI: 120 days	Burial conditions: Buried together in a shallow grave (30 cm), as well as a control burial. Environment: Burial site at Shanxi Medical University Summer to autumn	Sample: Soil sampled from 20 cm and 30 cm depth under femurs Control: Control samples collected from a control burial 1 m away; control soils were collected from 30 cm depth Sampling time: Days 0, 5, 10, 15, 20, 30, 60, 90, and 120	Dominant phyla in control and experimental soils: Actinobacteria, Proteobacteria, and Chloroflexi At family level Planctomycetaceae, Micrococcaceae, Sphingomonas, and Nocardioidaceae were dominant in experimental soils. The dominant taxa for control soils were Micrococcaceae, Sphingomonas, and Streptomycetaceae	Buried fresh bone has a unique microbial signature throughout the post-burial period, compared to control soils MAE of 55.65 ADD

**Table 4:** Summary of the methodological confounders and control measures in several studies applying microbial data from gravesoil.

Study	Sample collection and extraction	Sequencing design and bioinformatics workflow	Diversity analysis, statistical treatment, model handling
Cobaugh et al. (2015)	Handling: Samples stored at −20 °C until further analysis Extraction: PowerLyzer PowerSoil DNA Isolation Kit (Mobio Laboratories, Inc.)	16S rRNA gene V4 region Quantification: qPCR Primers: 1055F/1392R Primers: HuBac566f/HuBac692r Targeted for human-specific Bacteroides Platform: MiSeq (Illumina) Primers: 515F/806R Mothur (v.1.33.3) Pipeline: Chimaera removal (UCHIME) Taxonomic classification OTUs Taxonomic alignment (SILVA database) Normalisation: 121,340 reads per sample	Diversity and statistical analysis: Alpha Diversity: Simpson Diversity index and Chao richness ANOVA to measure statistical differences in microbial activity NMDS to visualise Bray–Curtis similarity between microbial communities
Weiss et al. (2016)	Handling: Soil probe cleaned with ethanol between samples; samples stored at −20 °C until further analysis Extraction: DNA extraction based on Earth Microbiome Project standard protocols and Metcalf et al. (2013)	16S rRNA gene V4 region Platform: HiSeq (Illumina) Primers: – QIIME Pipeline followed Metcalf et al. (2013) piprline: Pipeline alignment (Greengenes database)Normalisation: 14,000 reads per sample; additionally, also ran cumulative sum scaling (CSS)	Diversity and statistical analysis: UniFrac unweighted and weighted distances to explore abundances and patterns of community dissimilarity PERMANOVA for statistical significance of sampling groups based non-weighted and weighted distances
Olakanye et al. (2017)	Handling: Samples stored at −20 °C until further analysis Extraction: FastDNA1Spin Kit for Soil (MP Biomedicals, UK) Control: PCR negative controls	16S rRNA gene V4 region Platform: MiSeq (Illumina) Primers: Based on Kozich et al. (2013) (Kozich et al., 2013) Mothur Pipeline: Quality checked and filtered (UCHIME) Taxonomic alignment (SILVA database) Taxonomy assignment (RDP classifier) Normalised: 6750 sequences per sample	Diversity and statistical analysis: Alpha diversity: Shannon diversity ANOVA: TO evaluate all data Bray-Curtis (BC) distance un-weighted pair-group using arithmetic average (UPGMA) to test taxa similarities between the controls and treatments clustering algorithm Spearman’s rank correlation coefficient between soil pH, temperature and phyla relative abundance
Keenan et al. (2018)	Handling: Soil samples stored at −80 °C until extraction; extraction stored at −20 °C until further analysis Extraction: DNeasy Powerlyzer Powersoil kit (Qiagen)	qPCR Femto Bacterial DNA Quantification kit Targeted to human-associated *Bacteroides*	Statistical Analysis: One-way ANOVA: Differences between samples at depth and transects Two-way ANOVA: Effects of depth and distance along the transect PCA: overall differences in soil biogeochemistry between all samples
Olakanye and Ralebitso-Senior (2018)	Handling: Samples stored at −20 °C until further analysis Extraction: FastDNA1Spin Kit for Soil (MP Biomedicals, UK) Control: Triplicate extracts from control soil pooled	16S rRNA gene V1-3 region Platform: MiSeq (Illumina) Primers: 28F/519R Pipeline: Operational taxonomic unit selection (UPARSE) Chimaera removal (UCHIME) Taxonomy assignment (USEARCH) Phylogenetic tree generation (MUSCLE version 2.2.4)	Diversity and statistical analysis: Alpha diversity: Shannon diversity Phylogenetic distance matrices: Bray–Curtis dissimilarity with NMDS PERMANOVA (PAST 3.10, 2015): Differences at family-level taxonomic resolution between control and treatments (plant litter and pig) Pair wise multiple comparisons after a multi-way ANOVA for significant differences in OTUs between the control, treatments and seasons
Procopio et al. (2019)	Handling: Samples stored at −20 °C until further analysis Extraction: FastDNA1Spin Kit for Soil (MP Biomedicals, UK) Controls: PCR negative controls included	16S rRNA gene V4 region Platform: MiSeq (Illumina) Primers: 515FB/806RB Pipeline: Clustering into clustered into OTUs (VSEARCH v2.3.4) Taxonomic assignment (Greengenes v.13–8 database) Normalised: 38,684 sequences per sample	Diversity and statistical analysis: Alpha diversity: Shannon diversity index, Simpson index, Fisher index, Chao1, abundance-based coverage estimator Beta diversity: NMDS for visualisation of Bray-Curtis distances PERMANOVA to assess whether communities were statistically significant
Bisker et al. (2021)	Handling: Soil samples stored at −20 °C; extractions stored at −20 °C Extraction: FastDNA Spin Kit for Soil (MP Biomedicals, UK)	16S rRNA gene V4 region Platform: MiSeq (Illumina) Primers: 515F/806R QIIME2 Pipeline: Denoised (DADA2) Taxonomic assignment (Greengenes) Normalisation: 4000 samples per read	Diversity and statistical analysis: Kruskal-Wallis test: To determine significant differences in alpha-diversity PERMANOV: To test differences in beta-diversity
Burcham et al. (2021)	Handling: Soil samples transported to lab and stored at −80 °C Extraction: MoBio PowerSoil DNA extraction kit (MoBio Laboratories)	16S rRNA gene V4 region Platform: MiSeq (Illumina) Primers: 515F/806R QIIME2 Pipeline: Trimmed reads and denoised (Deblur v.1.1.0) ASVs creation and taxonomic assignment (Greengenes 13.8) Phylogentic tree generation (SEPP)	Diversity and statistical analysis: Alpha and Beta diversity: Shannon’s diversity, Pielou’s evenness, observed ASVs (richness), Faith’s phylogenetic diversity, and weighted and unweighted UniFrac distances PCoA for visualisation PERMANOVA: beta diversity metrics were analysed at 120 months, comparing the soil locations Machine learning algorithm: Random forest Validation:
Zhang et al. (2021)	Handling: Samples stored at −80 °C until further analysis Extraction: DNeasy PowrSoil Kit (Qiagen)	16S rRNA gene V3-V4 regions Platform: MiSeq (Illumina) Primers: 341F/806R QIIME Pipeline: Sequences merged, quality controlled, filtered and clustered (cutadapt, VSEARCH and USEARCH) Chimaera removal (UCHIME) Clustered into Operational Taxonomic Units (OTUs) Taxonomic assignment (SILVA (v132) database) Normalisation: 6982 sequences per sample	Diversity and statistical analysis: Alpha diversity: Shannon diversity index Bray-Curtis distance: Microbial community successions, community similarities PCoA based on Bray-Curtis distance: To visualise differences between samples of various time points PERMANOVA: to investigate the effect of PMI and sampling body sites on bacterial communities of burial cadavers Machine learning algorithms: Random forest Validation: 10-fold cross-validation
Cui et al. (2022)	Handling: Soil samples transported on ice to lab and stored at −80 °C Extraction: FastDNA Spin Kit for Soil (MP Biomedicals, UK)	16S rRNA gene V4 region Platform: MiSeq (Illumina) Primers: 515F/806R QIIME2 Pipeline: Read filtered, denoised, merged and chimaera removed (DADA2) Amplicon sequence variants (ASVs) creation and taxonomix assignment (Greengenes 13.8) Phylogentic tree generation (SEPP) Normalised: 21,310 sequences per sample	Diversity and statistical analysis: Alpha-diversity: Shannon and Chao 1 indices NMDS was used to determine the clustering of different soil samples based on the Bray–Curtis distance PERMANOVA was used to examine the difference in bacterial community compositions Redundancy analysis (RDA) was performed to arrange bacterial communities based on environmental factors One-way ANOVA with the Student–Newman–Keuls (SNK) test was used to compare the differences among samples Machine learning algorithms Random forest Validation: 10-fold cross-validation
Olakanye and Ralebitso-Senior (2022)	Handling: Soil samples stored at −20 °C Extraction: FastDNA Spin kits for Soil (MP Biomedicals, UK)	16S rRNA gene V4 region Platform: MiSeq (Illumina) Primers: SB701-702/SA501-508 Mothur (v.1.36.1) Pipeline: Sequences filtered and quality checked (UCHIME) Taxonomic classification (RDP)	Diversity and statistical analysis: Alpha diversity: Shannon–Wiener indices and Simpson diversity PCA was then applied to demonstrate temporal clustering and the differences in fungal and bacterial diversity Bray–Curtis dissimilarity with NMDS for phylogenetic distance matrices
Yang et al. (2023)	Handling: Samples stored at −80 °C until further analysis, and extraction stored at −20 °C Extraction: E. Z. N. A. Soil DNA Kit (Omega Bio-tek, Inc., Norcross, GA, USA)	16S rRNA gene V3-V4 regions Platform: MiSeq (Illumina) Primers: 341F/806R QIIME Pipeline: Reads quality controlled and filtered (Pear (v0.9.6), Vsearch (v2.7.1), and UCHIME) Sequences clustered into OTUs	Diversity and statistical analysis: Alpha diversity: Chao1, Shannon, and Simpson indexes PCA and NMDS for visualisation Machine learning algorithms Random forest Validation: 10-fold cross-validation
Bisker et al. (2024)	Handling: Soil samples transported on ice to lab and stored at −20 °C, extractions stored at −20 °C Extraction: FastDNASpin Kit for Soil (MP Biomedicals, UK)	16S rRNA gene V4 region Platform: MiSeq (Illumina) Primers: 515F/806R QIIME2 Pipeline: Denoised (DADA2) Quality filtered (UCHIME) Taxonomic assignment (RDP14 reference database)	Diversity and statistical analysis: Alpha diversity: Shannon diversity and Simpson index Kruskal-Wallis test was used to determine significant differences in alpha-diversity between groups. Two-way ANOVA for time and decomposition aboveground vs. in the subsurface
Wang et al. (2024)	Handling: – Extraction: FastDNA spin kit for soil (MP Biomedicals, UK)	16S rRNA gene V3-V4 regions Platform: MiSeq (Illumina) Primers: 341F/806R QIIME Pipeline: Clustered into OTUs taxonomix assignment (Greengenes 13.8) Normalised: 5631 sequences per sample	Diversity and statistical analysis: Alpha diversity: Shannon index Bray–Curtis distance: for differences in microbial community composition between groups of diversity Kruskal–Wallis test was used to test significant differences between burial and control soils. Spearman correlation analysis was used to evaluate the correlation between ADD and the relative abundance of each soil bacterial family Machine learning algorithms Random forest Validation: 10-fold cross-validation

In order to establish the PBI and PTI as a reliable framework for time-since-interval estimation, it needs to pass through the three categories of validation, which are development, internal validation and external validation to prove reliability (Budowle et al., 2008). This includes setting up empirically sound experimental proof-of-concept or pilot study designs to develop and optimise the PBI and PTI protocol for gravesoil, from collection to sequencing, as well as developing a regression-based model for predictive estimation. The field and laboratory protocol based on the framework in Figure 5, and predictive model can be validated internally by assessing its performance, sensitivity and reliability against control samples as well as additional empirical studies. Finally, the robustness of the entire framework can be validated externally through collaboration between independent laboratories. The validation process can involve two stages. The first stage can entail future research that contributes to building, testing and validating of such a predictive succession model using data from diverse geographic regions and burial conditions. The second stage can align with inter-laboratory proficiency where the framework’ and model performances are assessed independently by multiple laboratories. In so doing, the developed knowledge would contribute to the growing discourse of using microbial communities as a high-resolution and reliable tool in forensic investigations, particularly for use as a temporal indicator not only for time-since-death, but also the time-since-burial and time-since-translocation of a victim’s remains.

Lastly, complementing the validation process is a protocol that delineates essential information required for reporting (Table 5), thereby promoting reproducibility and standardization across studies. This template addresses the variability in methodological approaches and reporting of findings across current microbiome studies and aims to foster more inclusive and detailed reporting of key elements that form part of experimental designs from sampling to sequencing. Ultimately, the inclusion of the information highlighted in the table will allow for the advancement of forensic ecogenomics and use of soil microbial communities to aid PBI and PTI estimations, contributing to the admissibility and reproducibility within forensic science.

**Table 5:** Recommended structured reporting template outlining the essential information and methodological elements for reproducible, standardized and transparent forensic ecogenomics workflows.

Description	Key elements
Metadata	Site locations Case ID (if available) Date and time of site visits Site description Burial/deposition conditions Season Other multidisciplinary approaches incorporated
Sampling methodology	Sampling rational Sample site location Sample collection depth Sampling frequency Equipment used for samplingContamination controls Sterilization procedures
Sample handling	Conditions for sample storage and transportation from the field to the laboratory, as well as storage conditions at the laboratory
Controls and standards	Sampling: Inclusion of controls and field blanks during sample collection Microbial analysis: Positive and negative controls for the extraction and amplification process
MPS Analysis	Bioinformatics pipeline: Framework and version, plugins and packages (version number and year), reference database (version) Reporting of read depth, normalization procedures and validation Diversity analysis and statistical analysis
Data availability	Supplemental information Code or data storage and accessibility

The framework proposed in this review is modelled after established forensic microbial workflows to estimate physiological time (Pechal et al., 2014). The framework proposed by Pechal et al. (2014) is specifically designed for human-associated microbial succession. Although similar to the Pechal et al. (2014) framework, the analytical flow for the current proposed framework is adapted specifically for gravesoil-based samples and the PBI and PTI time-since-intervals being estimated. However, due to the shared focus on 16S rRNA gene profiling, there is a methodological overlap. By maintaining methodological continuity, the current framework allows for easier integration into existing or recommended forensic and post-mortem microbial clock workflows from sample collection to analysis, thereby contributing to a streamlined overall forensic workflow.

To aid in crime scene reconstruction, multidisciplinary empirical research are crucial for developing and refining novel and sensitive forensic methods for post-mortem time-since-interval estimation and clandestine grave location (Mansegosa et al., 2021; Berezowski et al., 2022). As a complementary approach, microbial data can also be integrated into multidisciplinary forensic workflows alongside forensic entomology (Iancu et al., 2015, 2016, 2018), forensic botany (Coyle et al., 2005; Wiltshire, 2009), drone-based remote sensing (Bodnar et al., 2019; de Bruyn et al., 2025) and geophysical approaches (Molina et al., 2015; Berezowski et al., 2022). The value-added outcome will be enhanced strength of the generated and collected data, and subsequent interpretation related to the temporality and treatment of the victims remains.

## 5 Laboratory and data analysis: biases and limitations

16S rRNA-based techniques are useful for characterising the microbiome of terrestrial ecosystems (Gkarmiri et al., 2017; DiLegge et al., 2022), aquatic ecosystems (Méndez-Pérez et al., 2020; Burtseva et al., 2021), the human body (Huttenhower et al., 2012; Kho and Lal, 2018) and for forensic analyses (Akutsu et al., 2012; Jesmok et al., 2016; Yang et al., 2024). Several challenges can, however, influence sequencing data, resulting in misrepresentation of the results, downstream interpretation and overall reliability of the derived PMI, PBI and PTI estimations. For instance, a considerable issue can be primer bias, where the primers do not align with the target DNA template to be amplified (Green et al., 2015). This can lead to distortions in the data as communities are either under- or over-represented in a sample (Lee et al., 2012; Poretsky et al., 2014; Silverman et al., 2021). During the sequencing run, it is also possible that cross-sample contamination can occur when indexes are misassigned to the wrong samples (sequences) due to barcode mismatching or index hoping (Guenay-Greunke et al., 2021). Additionally, a common problem in molecular laboratories is contamination of samples with low biomass input from shared reagents, equipment or workflows (Salter et al., 2014; Minich et al., 2019). Many of these challenges can lead to skewed results.

16S rRNA-based techniques are also limited by their reliance on the relative abundance of microbial communities (Poretsky et al., 2014). For microbial studies, raw sequence data are reported as proportions, as data are normalised by dividing counts for microbial features (OTUs or ASVs) by the total number of reads resulting in relative abundances (Zemb et al., 2020; Xia, 2023). However, the challenge of transforming counts to proportions to normalise data is that the observed microbial shifts are not necessarily reflective of the actual change in the total microbial community of the sample (Tsilimigras and Fodor, 2016). Instead, they could indicate compositional artefacts related to the expression of microbiome data as proportions normalised to a constant sum (Weiss et al., 2017; Alteio et al., 2021). While normalisation such as through rarefying (McMurdie and Holmes, 2014; Hong et al., 2022) is an important step in the bioinformatics workflow to correct for technical read depth or amplification biases, and to allow for the cross-sample comparisons, it can affect the reported microbial community composition (Weiss et al., 2017; Kumar et al., 2018; Swift et al., 2023). Apparent shifts in the relative abundance of one microbial community might be due to a decrease in the relative abundance of another microbial community, rather than reflecting biological change within the sample (Poretsky et al., 2014; Weiss et al., 2017; Swift et al., 2023). This can confound the results by obscuring increases, or overemphasising declines, as a portion of the data is removed (Tsilimigras and Fodor, 2016; Xia, 2023). Understanding the compositional bias within samples is important, especially when the total microbial load matters, such as in developing post-mortem microbial clocks for forensic investigations (Kaszubinski et al., 2020; Tozzo et al., 2022).

Transforming 16S data from relative to absolute abundances can be achieved through quantitative PCR (qPCR) (Zemb et al., 2020) or by for instance, cell counts through flow cytometry (Frossard et al., 2016; Vandeputte et al., 2017). When paired with appropriate internal standards, techniques such as qPCR (Dreier et al., 2022) and shotgun metagenomics (Poretsky et al., 2014) can complement 16S rRNA sequencing. By basing sequencing output on known quantities or absolute abundances, these approaches enable researchers to detect actual changes in microbial community abundance within samples (Durazzi et al., 2021). 16S rRNA, qPCR, and shotgun metagenomics are limited by the inherent variability of the experimental design and the preferred protocol for DNA extraction (Sui et al., 2020; Shaffer et al., 2022) and molecular microbial analysis (Schloss et al., 2011; Zhao et al., 2023). This inherent variability underscores the need for reliable absolute standards for reproducibility and comparison of sample data across biogeographic regions and time periods. Also, incorporating internal standards can aid in overcoming compositionality issues (Poretsky et al., 2014; Harrison et al., 2021). This is especially the case for interpreting shifts in microbial community abundance and diversity across different samples, time periods and environmental conditions. Spike-in control via the inclusion of a known amount of synthetic DNA to samples can help track the loss of DNA from the initial extraction, purification and amplification process (Poretsky et al., 2014; Tourlousse et al., 2016; Camacho-Sanchez, 2024). As part of good scientific practice and for quality control purposes, the inclusion of several controls in sample collection, processing and analysis is essential for veracity in forensic research. The inclusion of negatives and positive controls (Edmonds and Williams, 2018) and field blanks (Hornung et al., 2019) is useful to monitor contamination at different stages of the molecular analysis workflow, particularly in cases of outdoor field sampling. The inclusion of blanks during the extraction process can aid in further detecting any contaminated reagents in extraction kits (“kitome”) (Salter et al., 2014; Olomu et al., 2020). Ultimately, the controls incorporated into the workflow from sampling to analysis, including the potential contamination identified, should be reported transparently (Hornung et al., 2019).

Equally important is the appropriate use of machine learning approaches in microbiome research for forensic application to ensure they are scientifically sound and practical for real-world forensic cases. Currently, the limitations of machine learning for post-mortem time-since-interval estimation are that for datasets to be comparable, models are generated based on data from overlapping periods of decomposition, i.e., sample data from different studies are cut to the same decomposition timeline or post-mortem days. This means data from longer PMI and PBI periods are excluded from the datasets (Belk et al., 2019). Additionally, models are based on biases inherent in the dataset and experimental design, such as sampling site, project study period (weeks, months), environmental conditions, and molecular microbial analysis protocols (Metcalf, 2019; Namkung, 2020). As such, the same abiotic and biotic factors impacting the decomposition will also limit the application of machine learning models as universal predictive models (Chourasia et al., 2025). Because machine learning does not perform well at extreme ends of PMI (Belk et al., 2018), it is recommended that datasets need to be expanded to include microbiome data for extended post-mortem periods, applied further to prolonged post-burial and post-translocation intervals. The integration of machine learning into the development of PMI, PBI and PTI microbial clocks necessitates the standardisation of analytical protocols. Key methodological considerations include: cross-validation of data to prevent overfitting (Namkung, 2020) through holdouts, where machine learning models are trained on datasets, e.g., from specific sites, while withholding a single dataset such as a single site to validate the algorithm’s performance (Sharma et al., 2020; Papoutsoglou et al., 2023); and reporting of the variance of the model performance, i.e., sensitivity of the model’s predictions to changes in the training set (Ma et al., 2025; Romano et al., 2025).

To avoid inadvertently boosting model performance due to data leakage (Papoutsoglou et al., 2023), research and practitioner teams must ensure that, for example, temporally distinct samples from the same source (cadaver or gravesoil) are not unintentionally mixed into the training dataset. While the use of AI allows for the inclusion of large and complex datasets, their predictive models raise questions regarding generalizability and applicability to real-world forensic cases (Metcalf, 2019; Chourasia et al., 2025). Thus, further model testing is needed to capture more nuanced shifts in microbial communities after death for more reliable time-since-interval estimations across regions and seasons. Models need to be tested and cross-validated on diverse datasets including different burial conditions, different host models, different environmental conditions, and unknown training data to assess the generalizability of the machine learning models (Kubinski et al., 2022; Papoutsoglou et al., 2023), and to develop better predictive outcomes for post-mortem time-since-intervals using microbial data. However, there is currently a lack of complete and available datasets for PBI and PTI estimations, limiting their incorporation in machine learning algorithms to develop more reliable time-since-interval estimations. Finally, to make PMI, PBI and PTI results comparable and transferable between species and biogeographic regions, standardised protocols are needed to ensure the scientific rigour and robustness of data and the reproducibility and validity of results (Poussin et al., 2018; Schloss, 2018; Singh and Agarwal, 2024; Swayambhu et al., 2025), ultimately contributing to admissibility in forensic investigations.

## 6 Conclusion: challenges and future directions

Traditional methods, such as forensic entomology, forensic botany and forensic taphonomy, for estimating time-since-intervals in forensic investigations exhibit significant limitations due to the variability introduced by biotic and abiotic factors influencing the decomposition process. Additionally, these approaches rely largely on the experience and knowledge of the practitioner and the availability of regional databases for specimen identification (insects and plants), both of which are often lacking. Moreover, current experimental research designs fall short, often lacking replication and control burials, and failing to reflect current forensic casework. The characterization of the soil microbiome is a useful tool for clandestine grave identification. This review aimed to enhance the discussion related to post-mortem microbial clocks with an overview and introduction to the time-since-burial (PBI) and newly introduced concept of time-since-translocation (PTI).

This review recommends that microbial molecular ecology analysis through forensic ecogenomics offers a promising avenue for achieving accurate post-mortem time-since-interval estimations, encompassing PMI, PBI and PTI. Leveraging molecular approaches from ecology, we argue that forensic ecogenomics provides a viable tool to investigate clandestine burials through the analysis of shifts within gravesoil microbial communities for more precise post-mortem time-since-interval estimations. MPS and other molecular techniques, such as proteomics and transcriptomics have shown potential in characterising microbial communities, offering an innovative approach for reliable time-since estimations. Advancements in MPS have significantly enhanced our understanding of post-mortem microbial communities (thanatomicrobiome, epinecrotic microbiome, and soil microbiome) involved in decomposition. These microbial communities demonstrate considerable potential to be used as a universal microbial network for forensic applications.

The several examples presented in this perspective indicate that shifts in soil microbial communities for buried remains cannot only be used as a “microbial clock” to estimate the PMI. Instead, depending on the burial and environmental conditions, they can distinguish gravesoils months to years after deposition and burial. Additionally, the persistence of microbial communities in gravesoils is useful because it allows for the differentiation of gravesoils from equivalent undisturbed natural soils due to the presence of non-native bacterial taxa. This is useful not only for PBI estimation but also for locating clandestine graves. Emerging evidence from the reviewed studies indicates that the soil microbiome offers a useful tool that can contribute to post-mortem time-since intervals. The decomposition of a body leaves a lasting impression on the soil composition and microbial communities, which can persist from weeks to years depending on the burial conditions and the treatment of the body. For PBI and PTI estimations, this review identified bacterial phyla, Acidobacteria, Actinobacteria, Bacteroidetes, Chloroflexi, Firmicutes, and Proteobacteria, as consistent and informative biomarkers in burial contexts. At genus level, studies have reported that the presence or absence of specific microbial communities can be used to distinguish experimental (decomposition) soils from control soils without decomposing remains. However, a finer resolution, most likely species level characterisation is needed to distinguish plant litter from mammalian decomposition soils, and potentially animal from human derived decomposition. Additionally, considering that soil microbial communities undergo further shifts once remains are translocated, they could be useful in establishing a “microbial clock” for translocated remains. However finer taxonomic classification of microbial communities is needed for a more robust approach. By leveraging the changes in microbial community structure over time, forensic scientists can develop models to estimate both PBI and PTI.

This review highlights the need for further research to validate microbial community analysis across diverse biogeographical regions to enhance its precision and reliability as a tool for forensic investigations. Such validation could potentially improve the accuracy of post-burial interval (PBI) and post-translocation interval (PTI) estimations, ultimately enhancing methods for clandestine grave identification. To address the variability in reporting and methodological approaches across current microbiome studies, there is a need for standardisation and validation of experimental designs across diverse biogeographic regions and seasonal conditions to ensure broader applicability and reliability. Parallel with scenarios of surface depositions, future research should also focus on remains that have been buried in the sub-surface or relocated to refine and validate these models. To support standardization, transparency and reproducibility, it is recommended that methodological details and metadata related to the experimental designs, bioinformatics pipeline and machine learning protocol be included in future studies following community standards such as the Minimum Information about any (x) Sequence (MIxS) (Yilmaz et al., 2011). This review introduces a novel conceptual framework for PBI and PTI estimation alongside a reporting template. The reporting template outlines key information and methodological elements that must be systematically recorded and reported, including site metadata, sampling methodology, sample handling, the inclusion of controls and standards, microbial analysis and sequencing pipelines, and data availability. Along with standardised, reproducible and transparent outcomes, the recommended approaches will also allow for the cross-study comparisons and the inclusive integration of forensic ecogenomics into other multidisciplinary workflows. Integration of PBI and PTI estimation into the broader post-mortem time-since-interval estimations provides a more comprehensive approach, contributing to forensic investigations. The proposed conceptual framework, while still in the developmental stages, can contribute to and enhance ongoing efforts toward stringent practices and external validation for forensic acceptance. Ultimately, continued research and validation across diverse biogeographic regions are essential to establish forensic ecogenomics approaches as a standard practice, thereby enhancing the precision and reliability of forensic investigations, contributing to the resolution of crimes.
